# A duplex sequencing approach for high-sensitivity detection of genome-edited plants

**DOI:** 10.1016/j.fochms.2025.100278

**Published:** 2025-07-17

**Authors:** Laura Bonfini, Moreno Colaiacovo, Cristian Savini, Christoph von Holst, Matteo Maretti, Francesco Gatto, Federica Magni, Paloma Pérez-Bello, Davide Scaglione

**Affiliations:** a European Commission, Joint Research Centre (JRC), Via Enrico Fermi 2749, 21027 Ispra, VA, Italy; b Seidor S.r.l., via Castel Morrone 24, 20129 Milan, Italy; c European Commission, Joint Research Centre (JRC), Retieseweg 111, 2440 Geel, Belgium; d IGA Technology Services S.R.l., via Jacopo Linussio 51, I-33100 Udine, Italy

**Keywords:** Genetically modified organisms (GMOs), Detection, Duplex sequencing, *Solanum lycopersicum*, New genomic techniques (NGT), Next generation sequencing (NGS), Mutation

## Abstract

In this paper, we have evaluated a targeted high-throughput massive parallel sequencing approach for detecting single nucleotide mutations or small genomic changes generated by new genomic techniques (NGT).

We used unique molecular identifiers (UMIs) for the quantification of the mutant alleles and duplex sequencing to confirm a mutation on both strands to avoid polymerase chain reaction (PCR) artefacts or sequencing miss-calls. We tested the approach in blinded analyses on a set of mixed NGT-modified tomato lines and identified each single nucleotide mutation or small insert/deletion (InDel) down to a 0.1 % level. To our knowledge, this is the first performance evaluation of a duplex sequencing approach for detecting and quantifying small NGT DNA changes without a priori knowledge of the mutation type and position in a target region.

Our study advances the scientific discussion on detecting NGT-induced DNA modifications in plants and food products, evaluating the potential and current limitations of a cutting-edge NGS-approach.

## Highlights

• Detection of mutations generated by new genomic techniques (NGT) is challenging.
• We identified small NGT mutations using a targeted duplex-sequencing approach.
• Each mutation was quantified down to a 0.1 % level also in mixed samples.
• The approach does not require a priori knowledge of the exact mutation type.

## 1 Introduction

New genomic techniques (NGTs) can alter genomes with high precision at specified sites, often generating single nucleotide variants (SNVs) or short insertions/deletions (InDels) (Broothaerts et al., 2021). These small mutations, characteristic of genome-edited (GE) organisms and derived products, challenge the application of the current polymerase chain reaction (PCR)-based technology to the traceability and labelling of genetically modified food and feed products. Classical real-time PCR provides the adequate specificity and sensitivity for the identification and estimation of the relative abundance of conventional genetically modified organisms (GMOs) down to the 0.9 % (GM mass fraction) at ingredient level and to the 0.1 % level in feed as required by European Union legislation^3^^,^^4^. However, conventional GMOs have generally long transgenic sequences inserted into the host genome that can be targeted in the analyses.

New PCR analytical approaches have been recently employed (Peng et al., 2018, Peng et al., 2020) to screen and confirm the small mutations generated by NGTs or to evaluate the efficiency of mutagenesis protocols at specific sites. Such approaches have been applied also to the detection and quantification of genome edited (GE) organisms in mixed food or feed products. In those cases, droplet digital PCR (ddPCR) methods were able to provide high sensitivity, specificity and applicability for the detection and identification of SNVs (Fraiture et al., 2022; Zhang et al., 2021). Real-time PCR methods based on locked-nucleic-acid (LNA) technology have also been used for the detection and identification of a SNV in an herbicide-tolerant *Cibus canola* line (Chhalliyil et al., 2020) and for detecting a gene-edited site in rice (Zhang et al., 2021).

Relevant technical challenges come into play when exploiting the capabilities of real-time PCR at the limit of its potential to discriminate between a target sequence and an almost identical one. For instance, verification studies have indicated that the detection method for the *Cibus canola* NGT line is not able to distinguish unambiguously the target SNV from the non-mutated genomic locus and is therefore not suitable for GMO official control analysis (Weidner et al., 2022). Furthermore, the development of these methods requires prior knowledge of the mutation and of its sequence context and relies on ad hoc approaches depending on multiple technical factors. For example, it is not always possible to design in the target region PCR primers and probes with a suitable selectivity to unequivocally discriminate a single nucleotide variation introduced into a GE organism. PCR-based techniques have also limited discriminatory capacity for heterozygous or homozygous bi-allelic genotypes, since they are generally based on the analysis of relative fluorescence levels. Deviations from the expected 0 %–50 %–100 % frequencies may hamper correct dosage estimates by the clustering algorithms especially in polyploid species (Wang et al., 2021). Finally, real-time PCR and dPCR technologies cannot be designed to simultaneously detect many regions in the genome since multiplexing is usually limited to only a few targets, and compatibility of primers/probe sets must be verified (Grohmann et al., 2021).

A high-throughput target sequencing strategy may overcome these drawbacks. First, sequencing is a naïve approach: the information sampled from a whole genome, or a specific locus, is retrieved regardless of a priori knowledge of the mutation type and position. Secondly, by incorporating molecular barcodes or unique molecular identifiers (UMI) in genomic DNA fragments during library preparation, digital counting of molecules may provide a direct measurement, not mediated by signal intensity, of the frequency of a given mutation. Finally, the approach is amenable to the design of multiplex target sequencing, where hundreds of mutations can be simultaneously tested in the same sample or in multiple samples in parallel (Yasumoto & Muranaka, 2023).

High-throughput massive parallel sequencing approaches have been developed and validated in the biomedical field for the detection of low abundance sequence variants in cancer samples, in pathogens, as well as in other areas (Chen et al., 2023; Lanman et al., 2015; Newman et al., 2016). In the food and feed sector, these technologies have been explored for the detection of conventional GMOs (Arulandhu et al., 2018; Debode et al., 2019). The approaches have been also applied to the detection of products containing a well-characterised mutation (Fraiture et al., 2023), but not yet to the detection of products containing unknown mutations in putative target genes. This feature could be very relevant when there is no sequence information on the NGT product and, when possible, target genes could be identified according to the trait characteristics.

One major limitation of the sequencing approaches is the error rate, which could be, per se, too high for accurately confirming mutations at a low concentration level. In addition, all PCR-mediated enrichment steps used for the library preparation can introduce miss-incorporated nucleotides, even when using proofreading polymerases, which can be propagated in the amplicon populations and become undistinguishable from pre-existing low frequency mutations.

Some sequencing technologies may overcome such issues by using independent readings of the same original molecule. The amplification and sequencing errors can be identified and removed from individual sequence reads using the consensus of many reads bearing the same UMI (i.e. originating from the same molecule). Further distinction between PCR artefacts and true low frequency mutations in the template may be achieved by retrieving sequence information from both strands of an original template molecule (duplex-sequencing), as the probability of base miss-incorporation in both strands is close to zero (Schmitt et al., 2012).

In this paper we have evaluated a duplex sequencing strategy using UMIs in the library preparation for the detection, identification and quantification of products carrying analytically challenging genomic alterations such as SNVs or small InDels generated by NGT in a set of tomato target genes. Tomato (*Solanum lycopersicum L.*) is a suitable organism for testing the approach because it is a diploid species with a haploid set of 12 chromosomes and a small genome (950 Mb) (Hosmani et al., 2019). Moreover, tomato is a relevant crop species and fruit colour is an important horticultural trait, which greatly affects consumer preferences. Different genes in tomato have been targeted in NGT studies (Hashimoto et al., 2018; Jacobs et al., 2017; Kumar et al., 2024; Yang et al., 2023) and a GE modified tomato, which contains a single frameshift mutation in the glutamate decarboxylase (GAD) gene resulting in increased GABA accumulation, is also commercially available in Japan (Nonaka et al., 2017).

Our work provides a better understanding of the performance that may be achieved by using a targeted duplex sequencing approach for detection of short mutations at low frequency, especially when they are involving multigenic traits, and offers useful insights on possible acceptance criteria when using next generation sequencing (NGS) strategies.

## 2 Materials and methods

### 2.1 Experimental strategy

**Fig. 1:**
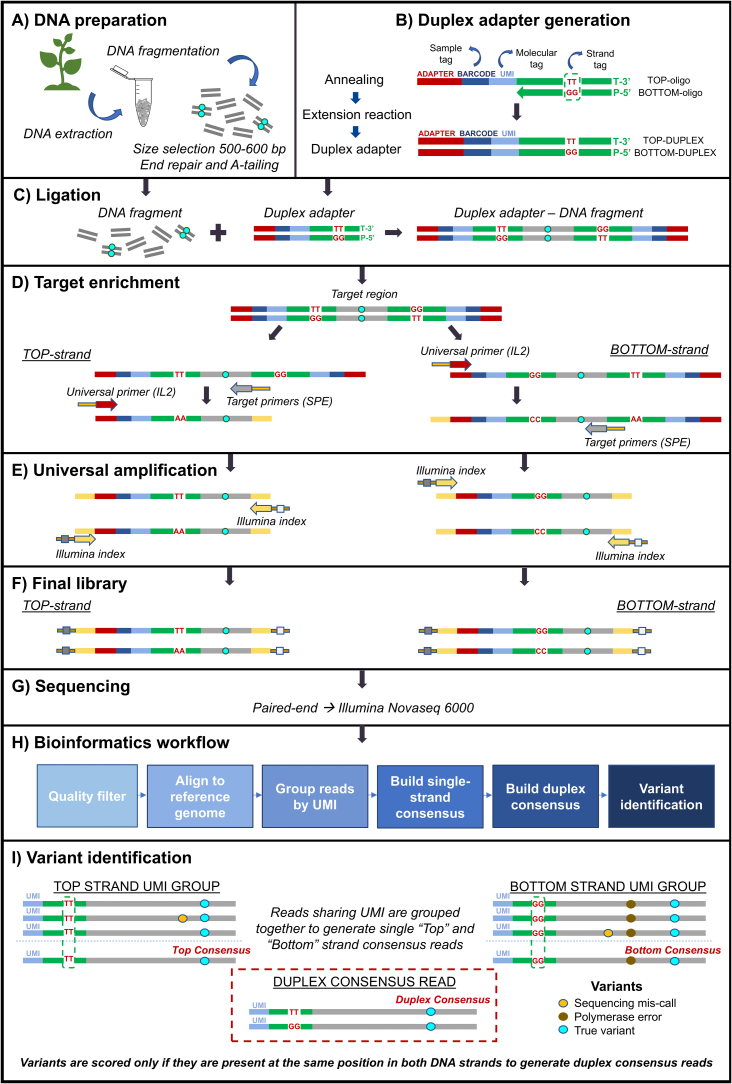
Duplex sequencing workflow. A) DNA preparation**:** Genomic DNA is extracted, mechanically fragmented, size-selected (500–600 bp) and subjected to end-repair and A-tailing. B) Duplex adapter generation: duplex adapters are designed to include an Illumina adapter sequence, a sample-specific barcode (sample tag), a Unique Molecular Identifier (UMI) to distinguish individual DNA molecules (molecular tag), and TT/GG nucleotides to differentiate the top and bottom strands (strand tag). The adapter preparation involves the annealing of the TOP and BOTTOM oligonucleotides, followed by an extension reaction to generate the duplex form of the adapter**.** C) Ligation: the DNA fragments produced in the first step (A) are ligated to the duplex adapters. D) Target enrichment: the target regions in the *Psy1* and *CrtR-B2* genes are amplified using a universal primer (IL2), which binds to the Illumina adapter sequence, and four gene-specific primers (SPE), that recognise the target sequences in the respective loci (PCR1). In the first cycle, the original TOP and BOTTOM strands are amplified with the SPE and the IL2 primers respectively. In the second cycle, the universal (IL2) and SPE primers amplify respectively their corresponding complementary strands. E) Universal amplification: Illumina P5 and P7 index adapters are inserted in the second PCR amplification step (PCR2) to allow Illumina paired-end sequencing of the target regions. F) Final library: final DNA fragments generated in the experimental procedure. G) Sequencing: paired-end sequencing is carried out on an Illumina platform (NovaSeq 6000). H) Bioinformatics workflow: the pipeline includes controls to filter out reads of low quality, alignment to the tomato reference genome, UMI-based grouping of reads, the generation of single-strand and duplex consensus sequences and high-confidence variant identification. I) Variant identification: the strategy involves the grouping of sequence reads sharing the same UMI, which originates from the same DNA molecule and the merging of complementary strands information, which can be distinguished by their TT and GG strand-specific tags. Single-strand consensus sequences generated from multiple reads with the same UMI allow to discriminate sequencing errors. Duplex consensus sequences generated from both strands of the original DNA molecule allow to distinguish polymerase errors introduced during the PCR amplification steps. Only variants present at the same position on both complementary strands are retained for variant calling.

We based our protocol development on the work by Peng et al., 2019. The rationale of this work, illustrated in Fig. 1, is to ligate custom-prepared adapters, carrying a duplex-specific unique molecular identifier (UMI) and a dinucleotide TT/GG marker of strand-specificity, to all the genomic DNA fragments generated during library preparation. A single locus-specific primer extension is then used to enrich for fragments carrying either the mutated or the wild-type target region without the necessity to design mutation-specific oligonucleotides. The tagged PCR products are further amplified and sequenced as paired-ends.

Fig. 1 illustrates also the variant identification strategy for differentiating mutations originally present in the genomic fragments from those generated during PCR amplification or sequencing. Reads are grouped in a strand-specific manner according to their UMI and used to generate a strand consensus sequence. A second consensus sequence is then produced across the two strands, masking with Ns the nucleotide positions with different bases on the two strands.

Fig. S1 in the supplementary Annex provides an illustration of the PCR enrichment step and the strategy for distinguishing reads derived from the same DNA strand or from the opposite strand of the original molecule.

### 2.2 Plant material

Seeds from NGT tomato (*Solanum lycopersicum*) lines and their wild-type counterpart were kindly provided by the Metapontum Agrobios Research Centre (Agenzia Lucana per lo Sviluppo e l'Innovazione in Agricoltura) under a material transfer agreement (MTA). These mutants were developed using the Clustered Regularly Interspaced Short Palindromic Repeats-associated protein 9 (CRISPR/Cas9) system by targeting the first exon of the genes B-Carotene hydroxylase 2 (CrtR-B2) and phytoene synthase (Psy1) each with two different constructs as described in D'Ambrosio et al., 2018. The modified knocked-down genes are involved in carotenoid biosynthesis and, when mutated, are easily detectable by a colour change: in the absence of CrtR-B2, petals turn white instead of yellow, while in the absence of Psy1 the ripening fruits turn yellow instead of red (Karniel et al., 2022). The NGT varieties selected for the experiments harbored SNVs or InDels within the first exon of these two genes (Table 1).

**Table 1:** Information on the NGT tomato lines used in the sequencing experiment. NGT tomato lines, their ID, corresponding mutated gene (CrtR-B2 or Psy1), region of interest (ROI), chromosome location (Chrom.), Start/End of the NGT mutant sequence, Type of NGT mutation and its Zygosity. Δ1 = line with one bp deletion, Δ5 = line with a five bp deletion, +1 = line with one bp insertion, ±1 = line with one bp deletion and one bp insertion on the opposite strands of the same locus.

NGT Line	ID	Gene	ROI	Chrom	Mutation Details			
					Start	End	Type	Zygosity
RS16#3–17-4	CrtΔ1	CrtR-B2	G03B2	SL4.0ch03	2,472,975	2,472,976	del1 bp (−C)	Homozygous
RS16#4–20-14	CrtΔ5	CrtR-B2	G05B2	SLA.0ch03	2,473,165	2,473,170	del5 bp (−CAGTT)	Homozygous
RS16#7–7	Psy+1	Psy1	G08PSY1	SLA.0ch03	4,235,352	4,235,352	ins1 bp (+A)	Homozygous
RS16#7–3-9	Psy_±1	Psy1	G08PSY1	SLA.0ch03	4,235,352 4,235,353	4,235,353 4,235,353	del1 bp (−A) ins1 bp (+C)	Heterozygous

Seeds of the NGT mutant and of a corresponding negative (azygous) transformed line (RS16#6–7) in *S. lycopersicum* cv Red Setter (here referred to as a wild-type line) were grown in a plant growth chamber (Aralab Fitoclima S600) for 28 days at the conditions specified in Table S1 (Supplementary Annex). Each growing cycle lasted 23 h and it was repeated approximately 29/30 times in the 28 days period.

### 2.3 DNA extraction and sample preparation

Sample preparation was conducted at the EURL GMFF laboratories of the Joint Research Centre. While the method used for this analysis is not under the scope of accreditation, the EURL GMFF is accredited under ISO/IEC 17025:2017 for the determination of GM content in food and feed using real-time PCR and digital PCR.

Tomato leaves (200 mg) were ground to powder in a mortar with liquid nitrogen following the manufacturer's protocol for the Biotecon Diagnostics DNA extraction kit (S 400 06.1), except for the final elution step which was carried out in 100 μL of TE buffer (0.1×; 10 mM Tris-HCl (pH 8.0) 0.1 mM EDTA). Multiple DNA extracts from the same tomato line were pooled into a single tube and stored at 4 °C until further use. All samples were analysed for their DNA quality and resulted to be compliant to the minimum acceptance criteria for DNA extraction of the European Network of GMO Laboratories (ENGL) (2015).

In particular, the presence of high molecular weight DNA observed via agarose gel electrophoresis confirmed the DNA integrity of the genomic extracts. None of the DNA samples presented an inhibition effect in the real-time PCR amplification tests conducted in accordance with Annex 2 in Hougs et al. (2017) and the ENGL guidance (2015).

The DNA concentration of each mutated and wild-type tomato line was measured in duplicates by fluorometric method using Invitrogen Quant-iT™ PicoGreen™ dsDNA Assay Kit (P7589) by Thermo Fisher Scientific. A five-point calibration curve was generated using different concentrations of a DNA calibrant and the respective fluorescence values (coefficient of determination (R^2^) resulted equal to 1.00 confirming the linear relationship between these two parameters). The DNA concentrations of the tomato wild-type and mutant lines were then calculated by interpolation from the calibration curve.

The correctness of the estimates was confirmed by real-time PCR experiments designed to target an unmodified region of the CrtR-B2 gene (Fig. S2). The same DNA amounts (100 ng) in the amplification reactions generated comparable threshold cycle (Cq) values, with a relative standard deviation equal to 0.5 % and PCR efficiencies all above 90 % (Tables S2, S3, S4).

Samples mixtures were prepared by spiking DNA from the NGT tomato lines CrtΔ1, CrtΔ5, Psy+1 into the genomic DNA of the wild-type azygous tomato line at a target NGT tomato content of 10 %, 0.9 %, 0.5 % and 0.1 % in DNA mass fractions. Given the source material (leaves) and the homozygosity of the CrtΔ1, CrtΔ5, and Psy+1 lines, these mass fractions correspond to DNA copy number ratios.

Because the heterozygous NGT line Psy ± 1 contains two edited alleles at the same site, DNA was mixed at double the mass used for the homozygous lines (CrtΔ1, CrtΔ5, Psy+1) to achieve target copy number ratio of 10 %, 0.9 %, 0.5 % and 0.1 % per allele. This adjustment ensured that the DNA mass fractions for Psy_±1 corresponded to the same copy number percentages as those for the homozygous lines.

Each mixture was then aliquoted into three 30 μL samples. For the 0.1 % level six replicates were prepared: three samples each were set up for using approximately 125 ng or 450 ng of tomato genomic DNA in the ligation with the duplex adapter. All the samples were submitted for library preparation and sequencing to IGA Technology Services Srl (IGATech). For the 0.1 % replicates, we provided details on the mutant allele's frequency, but not on the type of NGT mutation and relative position. For the remaining twelve samples including three replicates for the 10 %, 0.9 % and 0.5 % spikes and the wild-type lines we did not provide any information on the level, the position and type of NGT mutations in the target regions. Pure DNA samples of the spiked NGT lines were also submitted for sequencing to verify if additional sequence variations were present in the target genes.

All DNA samples were provided at a final concentration of 20 ng/μL.

### 2.4 DNA fragmentation, end repair and A-addition

Each genomic mixture (300 ng or 760 ng) was fragmented by sonication in 100 μL of 10 mM Tris-HCl pH 8.0 buffer using Bioruptor 300 (Diagenode) by applying three sonication cycles of 15” ON - 90” OFF. Each sheared mixture was size-selected using 0.8X AMPure XP beads from Agencourt and eluted in 50 μL of Tris-HCl pH 8.0 buffer. The average size (500–600 bp) of the recovered DNA was measured using Agilent Bioanalyzer High Sensitivity DNA chip, while the quantification was performed with Qubit dsDNA HS assay kit from Thermo Fisher Scientific.

Approximately 125 ng or 450 ng (for three 0.1 % samples) of the purified sheared DNA mixtures were end-repaired and A-tailed using KAPA Biosystems HyperPrep reagents (Roche) in a final volume of 60 μL ultrapure water following the manufacturer's instructions. The incubation conditions were 30 min at 20 °C and 30 min at 65 °C for the enzymatic inactivation. Each genomic DNA mixture was then used for the ligation reaction to the duplex adapter.

### 2.5 Duplex adapter

The generation of the Duplex adapter involves the annealing of the TOP and BOTTOM single-strand oligonucleotides (Table S5 of the Supplementary Annex), which is followed by an extension reaction to produce the double-stranded form. The TOP oligonucleotide includes the universal adapter sequence from Illumina (CCTACACGACGCTCTTCCGATCT) followed by the UMI sequences (represented as “NNNNNNNNNNNN”) and the sequences functioning as a template for the extension of the BOTTOM oligonucleotide. These sequences are complementary except for a two bp barcode (TT/GG) that allows the strand differentiation during the bioinformatics workflow.

### 2.6 Duplex-adapter preparation

The annealing reaction was performed by combining the TOP and BOTTOM single strand oligonucleotides at a final concentration of 30 μM and 60 μM respectively with 1× NEBuffer 2 (New England Biolabs) in a total volume of 12 μL. The oligo mixture was denatured at 94 °C for 5 min, cooled down to 21 °C at a rate no greater than 1 °C/min and finally kept at 4 °C. For the extension reaction, the annealed complex was combined with 1× Buffer 2 (New England Biolabs), deoxynucleotide triphosphate (dNTPs) at a final concentration of 0.5 mM each and 5 U of DNA Polymerase I (New England Biolabs) in a total volume of 20 μL. The reaction was incubated at 25 °C for 30 min and held at 4 °C. Purification of the duplex adapter was performed using MinElute PCR Purification kit (QIAGEN) specific for small DNA fragments (from 70 bp to 4 kb) following the manufacturer's instructions except for the elution in 25 μL 10 mM Tris-HCl pH 8.0 buffer. The size and purity of the duplex adapter were verified using Agilent 2100 Bioanalyzer High Sensitivity DNA assay (Agilent Technologies).

### 2.7 Ligation of the genomic fragments to the duplex-adapter

Duplex-adapter at a concentration of 0.02 μM presenting overhang thymidine bases was ligated to the A-tailed fragmented DNA aliquots in 110 μL final volume using KAPA Biosystems HyperPrep library preparation module kit (Roche) following the manufacturer's instructions. To ensure proper adapter ligation to DNA, the duplex adapters were added with a 20:1 M excess with respect to the A-tailed DNA fragments. A lower ratio could lead to DNA fragments not ligated to adapter sequences while an excess of adapter sequences could preferentially result in the formation of dimers that, due to their small size, could be preferentially amplified during the PCR- amplification step (Schmitt et al., 2012). The ratio between the adapters and the DNA fragments was calculated by using the molarity values obtained with the Bioanalyzer 2100.

Each reaction was incubated at 20 °C for 60 min, inactivated at 65 °C for 30 min and finally held at 4 °C. Ligated DNA complexes were purified from reagents and adapters excess using 1.2× AMPure XP beads (Agencourt) and eluted in 110 μL of ultrapure water. The size of the A-tailed and ligated DNA fragments (550–650 bp) was measured by using a high sensitivity DNA electrophoresis Bioanalyzer.

### 2.8 Primer design for target enrichment

A PCR-locus amplification step is achieved using a universal Illumina primer (IL-2) recognising a linker sequence in the duplex adapter and a set of four locus-specific primers (SPE) manually designed to anneal and extend as close as possible to two NGT target regions in both the Psy1 and CrtR-B2 genes. In this experiment, all four primers are designed to enrich the target NGT loci from the forward direction. The SPE primers include also a second Illumina adapter target sequence for the following sequencing reactions. Four SPE primers were designed according to the coordinates of the target loci of the NGT experiments (D'Ambrosio et al., 2018). The specificity of the four locus-specific primers has been evaluated using BLAT v.37 × 1 (-stepSize = 3 -tileSize = 8 -minScore = 20). No additional locations were predicted to align to the primers, besides the target regions in genes CrtR-B2 and Psy1. Using parameter -minScore 18 (instead of -minScore 20), for imperfect matches it was found only a match for 18 nucleotides out of 24 (75 % of the primer length) confirming the specificity of the primers. In Fig. S2 and Fig. S3 (Supplementary Annex), the target regions of the CrtR-B2 and Psy1 genes corresponding to the two candidate NGT loci carrying the mutations are highlighted in dark orange and blue colors. The respective SPE primers sequences are indicated in lighter colors.

The sequences of the IL-2 universal primer and of the SPE locus specific primers are provided in Table S6 (Supplementary Annex). The libraries purified from the first enrichment step were further amplified by PCR using custom dual-indexed oligos as indicated in Table S7 (Supplementary Annex).

All the oligonucleotides described in this study were synthesized by Integrated DNA Technologies (IDT). Lyophilized oligonucleotides were suspended in TE buffer (10 mM Tris, 0.1 mM EDTA, pH 8.0) to a final concentration of 100 μM and stored at −20 °C following the manufacturer recommendations.

### 2.9 PCR-NGT locus amplification

Duplex-ligated DNA aliquots were combined in ice in 50 μL total volume for the first PCR-locus amplification step (PCR1). The reagents and amplification conditions are described respectively in Table S8 and Table S9 of the Supplementary Annex. The enriched libraries (50 ng) were purified using 0.8× AMPure XP beads (Agencourt) and eluted in 25 μL 10 mM Tris-HCl pH 8.0 buffer. The libraries were then verified for their concentration with Qubit 2.0 Fluorometer DNA HS assay (Invitrogen) and for their size with Bioanalyzer High Sensitivity DNA assay (Agilent Technologies). The average size of the library after enrichment was about 500 bp.

About 200 ng of PCR1 product was combined on ice in 50 μL total volume for the second PCR-universal amplification step (PCR2). The reagents and amplification conditions are described respectively in Table S10 and Table S11 of the Supplementary Annex. A first bioinformatics analysis of the final sequencing data revealed for ten samples an over amplification of few template molecules. To solve the problem of poor UMI complexity the second PCR amplification was repeated with only 16 cycles instead of 20 at the same temperature and time conditions. Finally, all libraries were purified using 0.8× Agencourt AMPure XP beads, eluted in 25 μL 10 mM Tris-HCl pH 8.0 buffer, and verified by Qubit 2.0 Fluorometer DNA HS Assay (Invitrogen) and Bioanalyzer DNA assay (Agilent Technologies). All the reagents and relative company product codes are listed in Table S12 (Supplementary Annex).

### 2.10 Sequencing and bioinformatics data processing

The libraries were sequenced with 150 cycles in paired end mode (2 × 150 bp) on an Illumina NovaSeq 6000 instrument following the manufacturer's instructions (Illumina). The preparation of the libraries from the DNA samples of wild-type and NGT tomato lines, as well as from the mixtures of NGT lines at the different percentages, was performed by IGA Technology Services (Udine, Italy).The throughput for each sample at 0.1 % level was set to 100 million reads and for the remaining samples to 25 million reads.

An overview of the bioinformatics data processing workflow is illustrated in Fig. S4 (Supplementary Annex). For the minimal coverage study, a down sampling analysis was performed with the sequencing reads obtained from the 0.1 % libraries, using *seqtk* version 1.4-r122. From each of the 125 ng libraries, 25, 10 and 5 million read pairs were sampled; from the 450 ng libraries, an additional level of 50 million read pairs was considered. For each level and each library, three independent sampling replicates were obtained and analysed with the bioinformatics pipeline described in Fig. S4.

### 2.11 Statistical analysis

The statistical assessments have been conducted using the free software R, version 4.3.1 (R Core Team, 2023) via RStudio, version 2024.4.1.748 (Posit Team, 2024). The figures showing the results from the statistical assessment have been prepared using R package “ggplot2” version 3.4.3 (Wickham, 2016).

The mutation counts and read numbers in the results included values below 10, requiring the use of Poisson regression models, which are more suitable for this type of data. However, in the general discussion of the results we normalised the mutation counts to the number of total reads and expressed the values in the tables and figures as counts frequencies.

To examine the relationship between the number of reads and the mutation distance from UMI a Poisson regression model was fitted to the data, using the number of reads as response variable and the mutation distance from UMI as independent variable. For the latter variable, we utilised in the statistical analysis represented in Fig. S5 (Supplementary Annex) the midpoints of the bins containing a specific number of reads (e.g., the range 1–20 was represented by a midpoint of 10). Moreover, we considered only the first read in a pair (i.e. the one in proximity of the UMI adapter).

To identify the minimal coverage required for detecting the NGT mutations we evaluated the impact of the down-sampling level and the mutation type (independent variables) on the mutation counts. To analyse the data, we first established a Poisson regression model and then we applied a Chi-square test to assess the significance of the independent variables. We further included in the model the total number of reads as offset variable as specified in chapter 3.3. The whole data set comprised 180 counts (5 line-specific mutations 4 down sampling levels x 3 libraries x 3 replicates).

The relationship between mutation frequency and sample level, expressed as copy number ratio, was examined using quasi-binomial regression. The analysis was performed on the original, untransformed variables, which ranged from 0 to 1.

Model suitability was evaluated by examining the relationship between data variability and the expected value. Depending on the results, either a standard model (Poisson or binomial) or its extended form (quasi-Poisson or quasi-binomial) was applied. The choice reflected whether the observed variability aligned with the assumptions of the standard model.

To evaluate the performance of the duplex sequencing approach we examined two parameters: the precision, expressed as coefficient of variation (CV), and the trueness (bias) of the results. According to International Standard ISO 5725-2, 2019, the precision is the closeness of agreement between independent test results under specific conditions, while the trueness is the closeness of agreement between the average value obtained from a series of test results and the accepted reference values (here 0.1 %, 0.5 %, 0.9 % and 10 %). For GMO analyses the precision should be ≤ 25 % or ≤ 35 % for repeatability and reproducibility conditions respectively and < 50 % for relative concentrations < 0.2 % while the trueness should be within ≤ 25 % of the accepted reference value over the whole dynamic range (ENGL, 2015).

## 3 Results and discussion

### 3.1 Analysis of sequencing data for all tomato samples

Tomato DNA samples including wild-type, pure NGT mutant lines and 0.1 %, 0.5 %, 0.9 % and 10 % (in copy number ratio) mutant lines in wild-type background were submitted in triplicates for library preparation and sequencing. Initially ten samples showed skewed UMI distributions and low duplex read counts when using 20 PCR cycles in the first amplification step. To reduce overamplification, these samples were re-amplified using 16 PCR cycles. Sequencing confirmed accurate duplex sequence reconstruction without bias. Shapiro-Wilk test for normality (α = 0.05) and ANOVA revealed no significant difference (α = 0.05) in duplex read counts between the two library sets, allowing data aggregation. The final raw and processed sequencing data (alignments, duplex reads, UMI representation) obtained from the two library sets are displayed in Table S13 (Supplementary Annex).

The prepared libraries present a high enrichment specificity (total 93 %) with an average of 37 % and 56 % of the reads covering the CrtR-B2 and Psy1 target regions respectively. This is quite significant if we consider that the target regions are extremely small compared to the entire tomato genome. The libraries are also well represented since 27 % of the UMIs in single-strand consensus sequences correspond on average to 2–5 sequencing reads, and 15 % of them to more than 5 reads.

As shown in Table 2, the number of duplex reads is influenced by the amount of initial DNA molecules present in the ligation reaction. Therefore, a higher DNA input allows to capture extra molecules and to sequence those same molecules on both strands. A value of about 10 mean reads per UMI suggests that in our approach the same molecule was not over sequenced.

**Table 2:** Sequencing parameters for the duplex sequencing approach. Mean values for the levels 0.1 % at 125 ng ligation, 0.1 % at 450 ng ligation and the 0.5 %, 0.9 % and 10 % levels combined. a = mean number of single-strand consensus sequences in each library. At this stage, two ends of the same DNA molecule are considered as two separate consensus sequences. b = mean number of duplex consensus sequences in each library. At this stage two single-strand consensus sequences derived from two overlapping ends are merged into one duplex consensus if both ends are sequenced on both strands. c = mean number of duplex consensus sequences covering any of the target regions. d = mean percentage of the duplex consensus reads on target compared to the total number of duplex consensus reads. e = mean number of duplex consensus reads on target divided by the number of target regions (i.e. 4 regions). f = mean number of molecules that are sequenced at least on one strand, for each target region. g = mean number of reads associated to the same single-strand UMI. A very high number suggests that the same molecules are sequenced several times. h = mean number of duplex sequences covering each base of the NGT target region (i.e. a mean read depth of 1000 means that each targeted base is covered by 1000 different molecules sequenced on both strands).

Sample	0.1 %	0.1 %	0.5 %, 0.9 %, 10 %
Input amount	≈ 125 ng	≈ 450 ng	≈ 125 ng
Mean single stranded consensus reads ^a^	3,083,275	4,171,620	2,512,194
Mean duplex consensus reads ^b^	25,421	42,612	22,122
Mean duplex consensus reads on target ^c^	22,503	39,910	20,483
Mean on-target rate ^d^	89 %	95 %	93 %
Mean duplex consensus reads/primer ^e^	5626	9978	5121
Mean UMI/primer ^f^	493,090	658,228	409,578
Mean reads per UMI ^g^	9.65	12.20	10.31
Mean duplex depth ^h^	4945	8566	4482
Mean library concentration (ng/μL)	4.69	4.51	4.84
Mean insert size (bp)	199	148	165

Since the percentages of on-target reads were different for the CrtR-B2 and Psy1 genes (Table S13), we evaluated the parameter for each locus-specific primer (SPE). As indicated in Fig. S6 (Supplementary Annex), only 10–15 % of on-target reads is associated to the locus amplified by the CrtR-b2_2f primer, while about 20 %, 30 % and 35 % of on-target reads are associated to the loci amplified by the Psy1_1f, CrtR-b2_1f and Psy1_2f primers respectively. This suggests that the CrtR-b2_2f primer may have displayed a lower amplification efficiency in the PCR enrichment step.

### 3.2 Detection of mutations in the tomato target regions

**Fig. 2:**
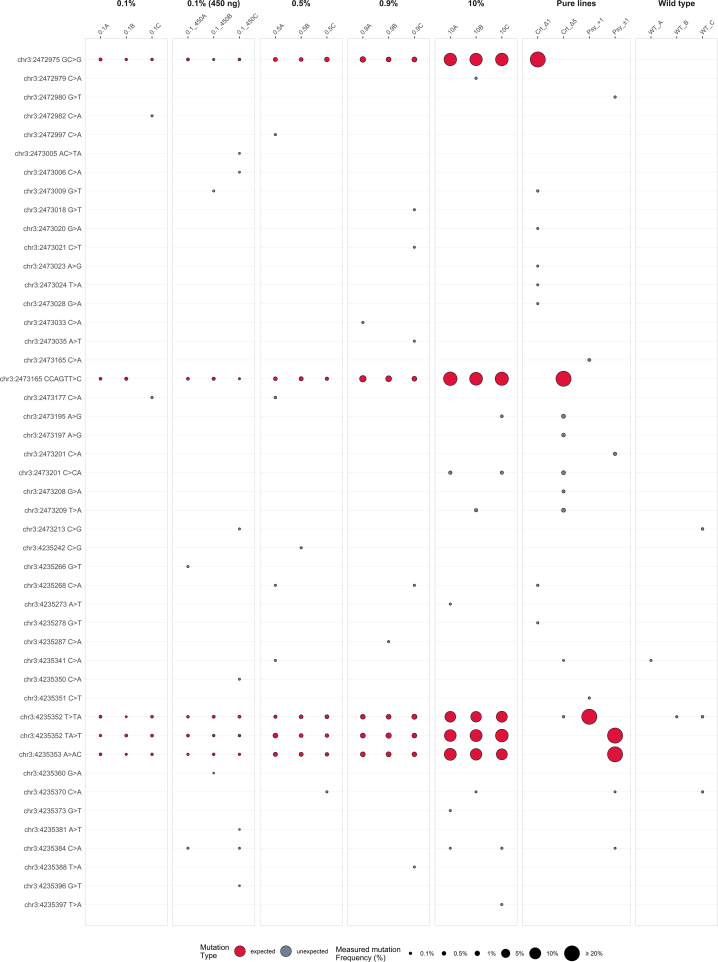
Bubble plot showing the mutations detected in the sequencing libraries and their frequencies. Expected mutations are plotted in red colour, while unexpected mutations are plotted in grey. The size of each bubble is proportional to the mutation frequency; for better clarity, all bubbles with frequency greater than 20 % have the same size. (For interpretation of the references to colour in this figure legend, the reader is referred to the web version of this article.)

We evaluated the mutations identified in the Psy1 and CrtR-B2 target regions for the different tomato samples at 0.1 %, 0.5 %, 0.9 % and 10 % fractions of spike-in edited DNA (see Supplementary Excel File S1 for the full data). As can be seen from Fig. 2, all expected NGT mutations are detected in all replicates and down to a minimum level of 0.1 % when 450 ng of genomic material are used in the ligation reaction, but not in all replicates when 125 ng of input DNA are alternatively used. These results substantiate the high sensitivity of the approach and suggest that an increase in DNA input may help detect mutations at very low frequency. It is noteworthy that the samples with 450 ng of template DNA in the ligation showed a much higher duplex coverage on average compared to the samples processed with 125 ng of DNA, as shown in Supplementary Excel File S1.

Some unexpected mutations are also identified in other locations of the same region at very low percentage (< 0.09 %) in only one or two of the three replicates and with similar estimated frequencies for all levels. This is consistent with a pattern of artefacts generated during library preparation, since they should occur with equal low probability in all samples, regardless of the level of the NGT lines. Only few unexpected mutations have been identified at a frequency higher than 0.1 % in the 10 % spiked-in samples. The same mutations have also been detected in the corresponding target regions of the pure NGT lines (Fig. 2 and Supplementary Annex Table S14) and are therefore probably pre-existing in the original samples. We performed bioinformatics analyses on these sequences and confirmed the absence of relevant PAM (protospacer adjacent motif) sites within three nucleotides of the unexpected mutations, supporting the conclusion that they were probably spontaneous mutations not generated by the genome editing process.

Sonication creates double strand breaks, and according to literature (Costello et al., 2013; Peng et al., 2019) also base damage errors that can be copied to the second strand during the end repair process, leading to base trans-versions (G-to-T from dG oxidation or C-to-T from dC deamination). During the fragmentation process, end-repair artefacts may also happen in the interior regions, but with a much lower frequency (You et al., 2020).

As shown in Fig. S5 (Supplementary Annex), we observed a significant trend of unexpected mutations to appear toward the end of the DNA fragments, as measured by a shorter distance to the UMI region in the reads, which was also supported by statistical analysis applying a Poisson regression model (*p* < 0.01). This is independent of the size distribution of the reads (see Supplementary Annex Fig. S7) and is consistent with artefacts generated by an end-repair mechanism after DNA sonication, similarly to the results obtained by Peng et al., 2019.

We also investigated whether the base substitutions detected in our samples followed a preferential pattern or if they equally concerned all bases and their possible variations. As illustrated in Fig. S8 (Supplementary Annex), the most common mutations in the raw data are T > A, C > A, and G > T substitutions. However, after the UMI single-strand consensus grouping, the error rates are heavily reduced and almost disappear at the duplex consensus stage. In fact, these artefacts are often supported by a minority of reads and are filtered out when establishing the single-strand consensus; if generated in the first amplification cycles, they are unlikely to be present on both strands. The duplex consensus possibly eliminates also mutations induced by sonication alone in the internal sequences of the genomic fragments, which are unlikely to be repaired and finally sequenced on both strands.

Mutations induced by sonication followed by end repair are present before the PCR amplification step and will not be filtered out by the duplex grouping. However, our data indicates that these mutations can be differentiated from those present in the original NGT lines for their low frequency (under 0.09 % in our dataset) and the fact that these errors, resulting from the random nature of sonication and end-repair mechanism, do not consistently appear across all replicates.

**Fig. 3:**
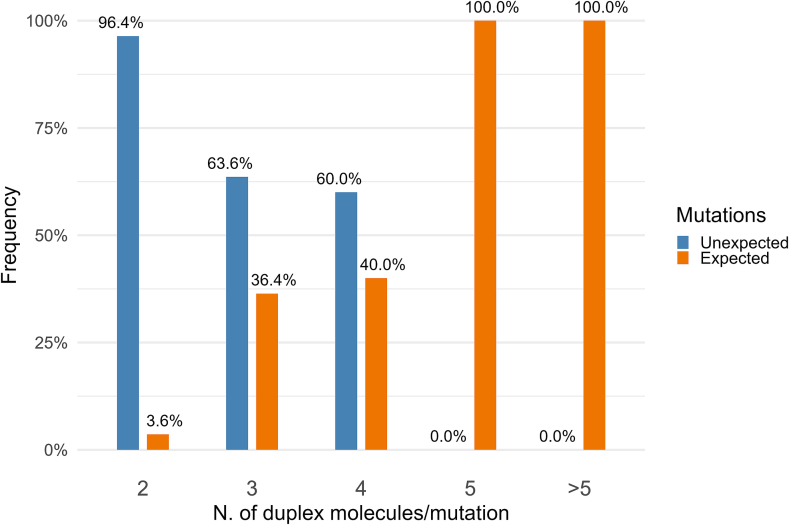
Frequency distribution of duplex molecules per unexpected or expected mutations. The mutations are grouped according to the number of duplex consensus reads supporting each mutation. For each group, the percentage of expected (orange) and unexpected (blue) mutations is reported. (For interpretation of the references to colour in this figure legend, the reader is referred to the web version of this article.)

To determine whether we could reduce the number of these variant artefacts, we investigated the distribution of duplex molecule counts for the expected and unexpected mutations. As indicated in Fig. 3, the sequence variants supported by only two duplex molecules correspond to non-expected mutations in 96 % of the cases. Conversely, the sequence variants supported by a higher number (five or more) of duplex molecules identify expected NGT mutations in 100 % of the cases.

To filter out false positive mutations we investigated by a heat map the optimal combination of duplex molecule counts versus number of replicates. The results are displayed in Fig. S9 (Supplementary Annex).

In the analysis we counted the number of expected/unexpected mutations detected for each combination of duplex counts/replicate counts (data in Supplementary Annex Fig. S10). We found a clear separation between expected and unexpected mutations at the combination four duplex counts / two replicates. Mutations called in three replicates are true NGT mutations in 100 % of the cases, while those called in two replicates can be considered as true positives only when they are supported by five duplex counts or more.

Overall, none of the expected mutations falls into the category of false negatives.

### 3.3 Minimal coverage analysis

To identify the minimal coverage required for detecting the NGT mutations at the 0.1 % level we performed a permutation/titration analysis. The datasets of the three 0.1 % samples (450 ng ligation) with a 50 million read coverage were analysed with the full number of sequenced reads, and in parallel with independent analysis of randomized subsampling of decreasing number of reads. The full pipeline was executed for 50, 25, 10 and 5 million fragments (i.e., read pairs), each with three independent replicate subsamples. The same analysis for the 0.1 % samples (125 ng ligation) was performed for 25, 10 and 5 million fragments (see Supplementary Annex Table S15 and Supplementary Excel File S2 for the full data). The mean mutant duplex counts from the three replicates at the different coverage levels were plotted versus the line-specific mutations, together with the total duplex counts and the estimated mutation frequency (Fig. S11 in Supplementary Annex). Visual inspection of the mutant duplex counts and total duplex counts across the various combinations of mutations and down-sampling levels, as shown in Fig. S11, suggested a correlation between these variables. To account for the potential influence of total duplex counts on mutant duplex counts, the Poisson regression model included the total number of reads as an offset variable. This model was fitted with mutant duplex counts as the response variable and down-sampling level and mutation as independent variables. The statistical analysis showed that the down-sampling had no significant impact on the mutant duplex counts (*p* = 0.63), whereas the effect of the mutation was highly significant (*p* < 0.01).

The data in Table S15 indicate that the raw sequencing coverage is not the limiting factor for detecting a mutation at the 0.1 % level, and that a coverage of 5 million reads could be sufficient for detecting mutations at very low concentration, provided a consensus is obtained from at least three replicates.

### 3.4 Quantification of mutations in the tomato target regions

To assess whether the duplex UMI sequencing approach could provide an acceptable estimate of the relative proportion of a mutant NGT sequence in a sample, we first evaluated if the mutation frequency was linearly dependent on the sample levels in the examined working range. Since one of the 0.1 % samples from the CrtΔ5 (hom) line prepared with 125 ng DNA input yielded a false-negative result, the subsequent statistical evaluation is based on the results obtained from the corresponding 0.1 % samples prepared with 450 ng DNA. At this input level, no false negative results were observed. The quasi-binomial regression analysis (Fig. S12) indicated for all mutants a linear relationship between the average mutation frequency and the sample level (expressed in copy number ratio).

**Fig. 4:**
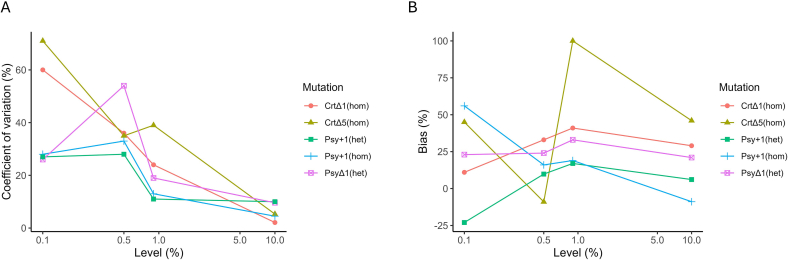
Plot of A.) the coefficient of variation (%) and B.) the bias (%) versus the target level (copy number ratio). Values on the X-axis are reported on a logarithmic scale to better visualize the variation across two orders of magnitude. Psy1+1 (het) and Psy_Δ1 (het) are two alleles of the same heterozygous line Psy_±1.

To evaluate the performance of the approach in more detail we examined two parameters: the precision, expressed as coefficient of variation (CV %), and the trueness (bias %) of the results. For the calculation of the CV and the bias, the relative abundance of the mutant allele (mutation frequency) was measured for each locus, as the ratio of the number of mutant duplex consensus sequences carrying the same exact mutation to the total number of duplex consensus sequences and averaged across the three library replicates (see Fig. 4 and Table S16).

In the working range examined the average CV values ranged from 6.3 % to 42 %. Comparison of the obtained values across the different levels and mutations reveals a clear trend with improving measurement precision at increasing concentrations, which is in line with the expected performance of analytical methods. Elevated CV values were observed particularly at very low levels (i.e., 0.1 %) (Fig. 4 and Table S16) where the quite small number (often below 10) of mutant duplex counts detected (see Excel S1 file) could explain the inherent variability predicted by the Poisson distribution. In addition, factors such as the sampling error and the variability associated to the sonication step and the measurement system can have an impact on precision, especially at these low levels.

At the legally relevant 0.9 % level, the CV values for the different mutations ranged from 11 % to 39 % and displayed an average of 21 %. This could be regarded as an adequate performance in comparison to the real-time PCR assays where CV values below 25 % are considered acceptable (ENGL, 2015).

Conversely, the bias resulted independent of all the levels and the mutations considered. Overall, the bias had an average value ranging between 15 % and 42 % for the different levels and from 2.5 % to 46 % for the different mutations with variable values across all levels and mutations. At the legally relevant 0.9 % level, the bias values ranged from 17 % to 41 % except for the CrtΔ5 (hom) mutation which displayed a particularly deviant value of 100 %.

Interestingly, the two mutant alleles of the heterozygous NGT line (one nucleotide insertion and one nucleotide deletion) presented different results. The Psy_+1 (het) mutation showed a good trueness over the dynamic range with a maximum negative bias of −23 % for quantification at 0.1 % and values ranging from 6.1 % to 17 % for quantification at the remaining levels (Table S16). The other allele, carrying the deletion (Psy_Δ1 (het)) with the same sequence context and library preparation samples, showed higher biases at each respective level (Table S16), suggesting a possible influence of the type of mutation on the reliability of its detection, which may be linked to sequencing chemistry or data processing issues.

It was not possible to identify a clear source for the variability of the bias values. Many different factors could be at play, including PCR amplification bias, library preparation, UMI complexity saturation and the specific bioinformatics approach being used (Peng & Dorman, 2023). Moreover, several studies have identified biases and errors resulting from library construction and sequencing chemistry on the Illumina instruments (Bullard et al., 2010; Dohm et al., 2008; Hansen et al., 2010; Li et al., 2010). Further work could explore the impact of these factors on performance parameters, for example by further optimising primers and UMI design, or by assessing different sequencing technologies and bioinformatics pipelines.

It is also important to note that the mutations were successfully identified with a significant effort (time, resources, and competences).

Currently the reagents used for our library preparation cost approximately 50 € per sample while the sequencing expenses can range between 130 € and 600 € per sample according to the multiplexing capacity of the platform and the experimental workload of the laboratory. These estimates do not consider labour and instrument amortisations.

The library preparation workflow can be executed in about four days (1 day in Peng et al., 2019), including library titration, pooling and loading while the sequencing step requires a 24-h run on an Illumina NovaSeq X Plus platform. A server node with 128 CPU and 512 GB of RAM can then perform the analysis of 10 samples in 48 h.

The whole library preparation process can be automated but this needs to be thoroughly validated, as precision in beads ratios is essential to maintain high-level template complexity during each step, while providing adequate removal of adapter artefacts.

The equivalent analyses by real-time PCR or digital PCR may still represent a cheaper and faster alternative for detecting GE organisms mutated at a unique site and for a longer sequence. However, analytical traceability of GE organisms presenting a short mutation, or multiple genetic alterations could be more challenging. It is not always possible to design in the target region PCR primers and probes with a suitable selectivity to unequivocally discriminate the variant sequence from the non-mutated counterpart (Gatto et al., 2023). Moreover, both real-time PCR and dPCR assays can currently target in a multiplex reaction only a limited number of genomic sequences (i.e. up to six).

The three technologies are compared for different performance parameters, costs and turnaround time in Table 3 (Bogožalec Košir et al., 2023; Grohmann et al., 2017; Lee et al., 2020; Morisset et al., 2013; Peng et al., 2020; Peng et al., 2019; Zhang et al., 2024). The values provided are only indicative since the cost is contingent on technical, logistical and geographical location, while the performance is dependent on many parameters such as the sample type, the objective of the analysis, or the target concentration.

**Table 3:** Comparison of duplex UMI amplicon sequencing, dPCR and real-time PCR with respect to multiplexing capability, analytical performance, DNA input requirement, cost and turnaround time. ^a^Peng et al., 2019; ^b^Bogožalec Košir et al., 2023. For the estimation of the turnaround time and relative cost/sample the testing process for qPCR included an initial screening with a 5-plex qPCR analysis followed by identification and quantification reactions on a 96-well plate; ^c^Grohmann et al., 2017; ^d^This study. The LOQ values 0.1 %–0.5 % for mutations Psy_+1 (het) and Psy+1 (hom) respectively, correspond to values of CV % below 35 % and bias below 25 %; ^e^(Morisset et al., 2013). The turnaround time is calculated from pipetting the premade reaction mixes on a 96-well plate to the analysis of the results; ^f^(Lee et al., 2020); ^g^(Peng et al., 2020); ^h^(Zhang et al., 2024). n.r. = not reported; log = logarithmic scale.

Parameter	Duplex UMI amplicon sequencing	dPCR	qPCR
Multiplexing	Up to 192-plex^a^	Up to 6-plex^b^	Up to 6-plex^c^
Limit of Detection (LOD)	0.1 % (450 ng)^d^	< 25 copies^b^ 5–6 copies^e^	1–10 copies^e^
False negative rate	0 % at 0.1 % (450 ng)^d^ 3.4 % at 0.2 % (160 ng)^a^	n.r.	n.r.
False positive rate	0 % at 0.1 % (450 ng)^d^ 0 % at 0.2 % (160 ng)^a^	0 %^b^	n.r.
Dynamic range	0.1 %–10 %^d^	5 logs^e^	5 logs^e^
Limit of Quantification (LOQ)	0.1 %–0.5 %^d^	12–47 copies^b^ 5–18 copies^e^	18–60 copies^e^
Precision (CV%)	6.3 %–49 %^d^	< 15 %^b^ < 25 %^e^	< 35 %^e^
Trueness (bias %)	15 %–42 %^d^	0.23 %–15 %^b^ −9 %-10.4 %^e^	−24.7 %–11.1 %^e^
DNA input requirement	160 ng^a^ 450 ng^d^	9 ng^e^	50 ng^e^
Cost/sample	130 €-600 €^d^	1X^b^ 16.1$^e^	2.94X^b^ 23.3$^e^
Turnaround time	4 days^a^ 7 days^d^	3.5 hours^b^ 6 hours^e^	16 hours^b^ 3 hours^e^
Strengths^f,g,h^	High throughput method High multiplex capacity High sensitivity and specificity	High sensitivity and specificity Cheaper than duplex sequencing Absolute quantification (No need for CRMs) Less prone to inhibition	Wide dynamic range Cheaper than duplex sequencing Fast and easy to perform than dPCR and duplex sequencing
Weaknesses^f,g,h^	Time consuming More costly Bioinformatics expertise required Higher DNA input	Low multiplex capacity Low throughput method Limited dynamic range Extra step for droplet generation	Low multiplex capacity Extensive optimisation for multiplex methods Standard curve required Higher false positive and negative rate than for dPCR

## 4 Conclusions

The EU legal framework on GMOs requires the provision of a detection method in the application for authorisation to ensure the traceability of GMOs and the enforcement of the labelling requirements. Conventional GMOs are detected and identified even at low copy-number and in complex matrices by PCR-based methods targeting the unique plant-to-transgene junction. Conversely, the detection of the mutated site in edited organisms can be more challenging as the only genetic information that can be targeted lies in the few altered nucleotides. Moreover, PCR-based detection depends crucially on the prior knowledge of the mutation and its sequence context.

High-throughput sequencing strategies may overcome these drawbacks by providing information from a whole genome or a specific locus regardless of a priori knowledge of the mutation type and position. Secondly, by incorporating unique molecular identifiers (UMI) in genomic DNA fragments during library preparation, digital counting of molecules may offer a direct measurement of the frequency of a given NGT mutation. Finally, a duplex sequencing approach can remove PCR amplification and sequencing errors even at the concentration level required by EU legislation (at or below 0.9 %).

As a proof of concept, we have tested this approach on tomato lines generated by CRISPR/Cas9 technology that presented different types of mutations in specific loci and genes.

The strategy has been very effective in detecting the expected NGT mutations in all three replicate samples and independently of the type of sequence variation. We were able to detect the mutations down to 0.1 % in all NGT lines when the ligation reaction for the preparation of the libraries contained 450 ng of input DNA, or in most of the NGT lines when using a 125 ng DNA input. A higher DNA input can therefore be beneficial not only to capture other different molecules, but also to sequence those molecules on both strands. As a rule, we also suggest adjusting the amount of DNA used in the preparation of the libraries to the genome size, since the number of template copies can be significantly lower with large genomes. Given our results, at the very minimum we recommend using 100 ng per 1 Gbp (i.e., 1 billion base pairs) of haploid genome size of the organism being studied.

This approach allowed also to test multiple NGT-induced mutations in the same sample. This may help identify GE plants when variety-specific markers are known to be associated to the NGT mutation or identify multiple mutations on the same genome.

We have detected some unexpected mutations at very low level in only one replicate and very rarely in two of the three replicates of some sequenced samples. By adopting filtering criteria based on a minimum of two duplex consensus reads in three independent replicate samples, we could retain only the expected NGT mutations, irrespective of their prior knowledge. However, other filtering strategies could be alternatively applied in this or other contexts as suggested in Fig. S9.

To identify the minimal coverage required for detecting these mutations at the 0.1 % level we performed a permutation/titration analysis for 50, 25, 10 and 5 million reads, each with three independent replicate subsamples. The analysis indicated that the subsampling does not significantly affect the quantification at the 0.1 % level and that this result is similar for all mutation types. This suggests that it may be possible to reduce the amount of reads and costs required for sequencing analysis, without affecting the sensitivity of the results.

Acceptance criteria for trueness and precision in NGS experiments measuring the relative abundance of short NGT mutations are not established. Therefore, we have assessed the performance of the duplex sequencing approach considering the acceptance criteria established by the ENGL, 2015 for qPCR GMO analysis. We acknowledge however, that the two technologies have different concepts and target detection challenges.

The trueness (bias) values of our sequencing approach were in some cases higher than 25 % in the dynamic range being tested, while the CV % values were below the 25 % limit only at the 10 % and 0.9 % levels (except for one NGT mutation at 0.9 %). In our study, we expected to measure values above a threshold of 25 %, notably at the lower end of the spiked range. Indeed, the challenges of detecting and quantifying short mutations, such as those induced by gene editing, are not comparable to those for ordinary GMOs where long foreign DNA sequences are introduced in an organism.

Interestingly, at the 0.9 % legal threshold for GMOs, our study found that the duplex sequencing approach could accurately quantify certain types of mutations, fulfilling the acceptance criteria for accuracy established for PCR-based methods. However, this was not consistently observed across all lines. Consequently, no general conclusion can be drawn at this stage, and any potential application of this methodology should be experimentally assessed.

To our knowledge, this is the first time a duplex sequencing method has been used to detect single nucleotide variants in NGT plants without prior information about the mutation type. Moreover, the sequencing provider was not given any details about the level and position of the NGT mutation in the target regions; instead, the analyses were conducted on all detected mutations. This approach could be especially useful when a new phenotype suggests the presence of an NGT plant as it allows to verify the sequences in the relevant gene or multigenic pathway without needing to know the exact mutation. Our work addresses the analytical and quantification challenges posed by short DNA alterations induced by NGT and provides a better understanding of the performance that may be achieved for detection of short mutations at a low frequency, especially when they are involving multigenic traits. It finally offers useful insights on possible acceptance criteria for NGS strategies. Based on our findings and limited to the constraints already discussed, we show that the acceptance criteria established for qPCR (ENGL, 2015) for parameters such as trueness and precision are not consistently met by a sequencing approach. In the current investigation, the trueness was quite small at least for some mutant lines (1 bp insertion) but much larger in other lines, and the overall variability decreased as expected at higher concentration levels. Method optimisation can improve the accuracy of the results.

It is also important to note that the mutations were successfully identified with a significant effort in the frame of a research project, and that a routine implementation of this approach could be challenging. However, the implementation of gene editing for improving multigene-controlled traits in crop cultivars may reduce the cost and increase the feasibility of NGS analyses with respect to real-time PCR and dPCR approaches.

Further to initial testing, additional research is necessary to assess the appropriateness of this approach. Indeed, mutation identification, low level detection, transferability to different contexts, all require proper optimisation, standardisation, as well as the acquisition of knowledge from experimental evidence for scientific advice. In addition, it is not well established if all types of short DNA alterations could be detected at low levels in different DNA contexts and with different primer efficiency. Finally, the detection of a short mutation is not sufficient, per se, to determine its origin, whether by natural processes, conventional breeding or gene editing (Grohmann et al., 2019; Gatto et al., 2023; ENGL, 2023).

## CRediT authorship contribution statement

**Laura Bonfini:** Writing – review & editing, Writing – original draft, Validation, Supervision, Project administration, Funding acquisition, Conceptualization. **Moreno Colaiacovo:** Writing – review & editing, Visualization, Validation, Investigation, Formal analysis, Data curation, Conceptualization. **Cristian Savini:** Writing – review & editing, Validation, Funding acquisition, Formal analysis, Conceptualization. **Christoph von Holst:** Writing – review & editing, Visualization, Validation, Formal analysis, Conceptualization. **Matteo Maretti:** Resources, Investigation. **Francesco Gatto:** Writing – review & editing, Funding acquisition, Conceptualization. **Federica Magni:** Investigation. **Paloma Pérez-Bello:** Writing – review & editing, Visualization, Data curation. **Davide Scaglione:** Writing – review & editing, Visualization, Validation, Resources, Methodology, Formal analysis, Data curation, Conceptualization.

## Declaration of competing interest

The authors declare the following financial interests/personal relationships which may be considered as potential competing interests: Davide Scaglione and Federica Magni report financial support was provided by European Commission Joint Research Centre Ispra. Moreno Colaiacovo reports administrative support, article publishing charges, equipment, drugs, or supplies were provided by European Commission Joint Research Centre Ispra. Moreno Colaiacovo reports a relationship with European Commission Joint Research Centre Ispra that includes: non-financial support. Moreno Colaiacovo reports a relationship with Seidor srl that includes: employment. Paloma Perez-Bello Gil reports financial support was provided by Programma operativo del Fondo Sociale Europeo (FSE) della Regione Autonoma Friuli Venezia Giulia. Paloma Perez-Bello Gil reports a relationship with Programma operativo del Fondo Sociale Europeo (FSE) della Regione Autonoma Friuli Venezia Giulia that includes: funding grants. If there are other authors, they declare that they have no known competing financial interests or personal relationships that could have appeared to influence the work reported in this paper.

## Data Availability

The link to the dataset has been shared in the submission process and mentioned in the manuscript European Nucleotide Archive (ENA) at EMBL-EBIA duplex sequencing approach for high-sensitive detection of genome edited plants (Original data)

## Glossary

bp: base pair
CRISPR/Cas9: Clustered Regularly Interspaced Short Palindromic Repeats-associated protein 9
CrtR-B2: B-Carotene hydroxylase 2
CV: Coefficient of Variation
dsDNA: double-stranded DNA
duplex sequencing: library preparation and analysis method for next-generation sequencing (NGS) platforms that employs random tagging of double-stranded DNA to detect mutations supported by both strands, ensuring higher accuracy and lower error rates
EDTA: Ethylenediaminetetraacetic acid
GAD: glutamate decarboxylase
GE: genome-edited
GM: genetically modified
GMO: genetically modified organisms
LNA: locked-nucleic-acid
InDels: insertions/deletions
NGS: next-generation sequencing
NGT: new genomic techniques
dNTPs: deoxynucleotide triphosphate
PCR: polymerase chain reaction
ddPCR: droplet digital PCR
Psy1: phytoene synthase 1
ROI: region of interest
SNV: single nucleotide variants
SPE: locus-specific primer
UMI: unique molecular identifiers
WT: wild type

## Appendix A Supplementary data

Supplementary material 1 Image 1

Supplementary material 2 Image 2

Supplementary material 3 Image 3

## Footnotes

3 Regulation (EC) No 1830/2003 of the European Parliament and of the Council of 22 September 2003 concerning the traceability and labelling of genetically modified organisms and the traceability of food and feed products produced from genetically modified organisms and amending Directive 2001/18/EC. OJ L 268, 18.10.2003, p. 24-28. This legislation requires mandatory labelling of GM food and feed products at a concentration higher than 0.9% (GM mass fraction) at ingredient level.

4 Commission Regulation (EU) No 619/2011 of 24 June 2011 laying down the methods of sampling and analysis for the official control of feed as regards presence of genetically modified material for which an authorization procedure is pending or the authorization of which has expired. *OJ L 166, 25.6.2011, p. 9–15.*
