# Supplementary material for: A duplex sequencing approach for high-sensitivity detection of genome-edited plants
Source: Food Chem (Oxf). 2025 Jul 17;11:100278. doi: 10.1016/j.fochms.2025.100278 (PMC12312065; doi:10.1016/j.fochms.2025.100278)
Supplement: Supplementary file 1 — Supplementary material 1 [file mmc1.docx]

**Supplementary Annex**


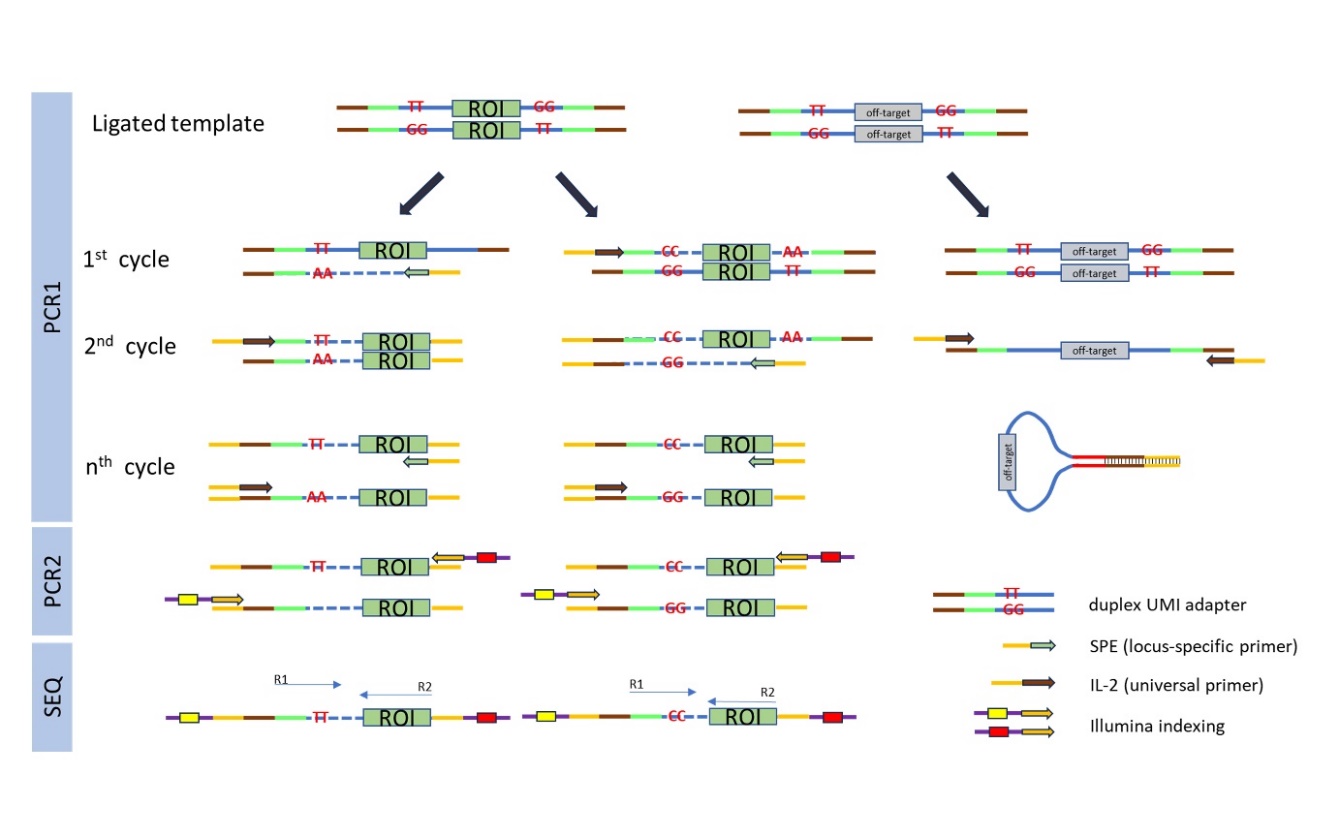


**Figure S1.** PCR enrichment and Duplex adapter strategy. A duplex adapter containing a double-stranded UMI (green) is attached to each side of a template molecule. In the first PCR amplification step (PCR1) for target enrichment a molecule containing the region of interest (ROI) is primed by the locus-specific primer (SPE) extending a complementary fragment while including an Illumina oligo tail (yellow). A universal primer designed on the duplex adapter linker (brown) will always prime on the duplex adapter. A target locus will benefit from an exponential amplification of relatively shorter fragments. An off-target molecule can be amplified by means of the universal primers on both ends but is impaired by the PCR suppression mechanism of complementary ends (Dai et al., 2007) and by the relatively lower performance of a bigger insert size. Illumina indexing and completion of the functional library is carried on the second PCR amplification step (PCR2) with directional oligonucleotides. The sequencing of a library fragment derived from a top strand of ligated template will provide in the first PCR1 step a molecule specific UMI and a TT barcode, while a fragment generated from the same molecule but amplified from a bottom strand will sequence the same UMI but with a CC barcode. The PCR2 amplification step will provide most of the coverage for the sequencing (SEQ) of the region of interest (ROI).

| **Segment** | **Time: minutes** | **Temperature: °C** | **Humidity: % RH*** | **Light Power: %** | **Ventilation: %** |
| --- | --- | --- | --- | --- | --- |
| 1 | 480 | 18 | 70 | 0 | 70 |
| 2 | 30 | 25 | 70 | 10 | 70 |
| 3 | 840 | 25 | 70 | 100 | 70 |
| 4 | 30 | 18 | 70 | 10 | 70 |

**Table S1.** Conditions used for growing the seeds of the tomato lines. RH*= relative humidity

| **Sample code** | **R^2^ coefficient** | **Amplification Efficiency** | **Mean Cq** |
| --- | --- | --- | --- |
| Unmodified line | 1.00 | 96 | 21.4 |
| RS#3-17-4 | 1.00 | 94 | 21.3 |
| RS#4-20-14 | 1.00 | 92 | 21.4 |
| RS#7-7 | 1.00 | 95 | 21.4 |
| RS#7-3-9 | 1.00 | 92 | 21.2 |
| Mean Cq (across lines) |  |  | 21.4 |
| RSD % |  |  | 0.53 |

**Table S2.** R2 coefficient, amplification efficiency and mean quantification cycle (Cq) values of the tomato CrtR-B2 amplification assay. Genomic DNA was extracted from the wild type and the mutant tomato lines. The reactions were performed in duplicates from the undiluted sample (at 100 ng DNA per reaction) and from a 1.4 five-point serial dilution. Cq: threshold/quantification cycle; RSD: relative standard deviation.

| **Oligonucleotide** | **Sequence (5’=> 3’)** | **Final concentration** |
| --- | --- | --- |
| Forward primer | TTTCAGCCTCCGCTAGTT | 400 nM |
| Reverse primer | TGCGAGTTAACGGAGAGAA | 400 nM |
| Probe | HEX-AG+TC+CAAAAT+CC+GCC-IowaBlack | 200 nM |

**Table S3.** Primers and probes for the tomato CrtR-B2 amplification system. The reaction was conducted in a final volume of 25 µL with the Universal Master Mix (Cat number 4318157). + denotes the position of LNA nucleotides.

| **Step** | **Stage** | | **T (°C)** | **Time (s)** | **Acquisition** | **Cycles** |
| --- | --- | --- | --- | --- | --- | --- |
| 1 | UNG | | 50 | 120 | No | 1 |
| 2 | Initial denaturation | | 95 | 600 | No | 1 |
| 3 | Amplification | Denaturation | 95 | 15 | No | 45 |
|  |  | Annealing & Extension | 60 | 60 | Yes |  |

**Table S4.** Cycling program for the tomato CrtR-B2 amplification system. In the first step, an Uracil-DNA Glycosylase treatment is carried out at 50°C for 2 minutes followed by a denaturation step at 95°C for 10 minutes. In the third step, a denaturation at 95°C for 15 seconds and an annealing/extension at 60°C for 1 minute are repeated for 45 amplification cycles during which data is acquired.

| **Adapter ssDNA** | **Sequence** | **Length (bp)** |
| --- | --- | --- |
| TOP | 5′ -/5InvddT//iisodG//iisodG/**CCTACACGACGCTCTTCCGATCT**NNNNNNNNNNNNTTCTGAGCGATTATAGGAGTCC*T - 3′ | 58 |
| BOTTOM | 5' - /5phos/GGACTCCTATGGTCGCTCAGAA - 3' | 22 |

**Table S5.** Duplex adapter sequences. The top and bottom oligonucleotides are used for the preparation of the adapter. The sequence highlighted in purple is annealed by Illumina universal primer IL-2 in the first PCR locus amplification step. Red mismatches TT/GG are those providing strand information on UMI counts. 5InvddT: Inverted Dideoxy-T at the 5’ end of a sequence prevents unwanted 5’ ligation and nuclease degradation; iisodG: guanin isoform; (*) Phosphorothioate bond: gives resistance to 3’ degradation by some 3’-exonucleases and blocks extension by DNA polymerases; 5phos: 5’ Phosphorylation is needed when an oligo is used as a substrate for DNA ligase.


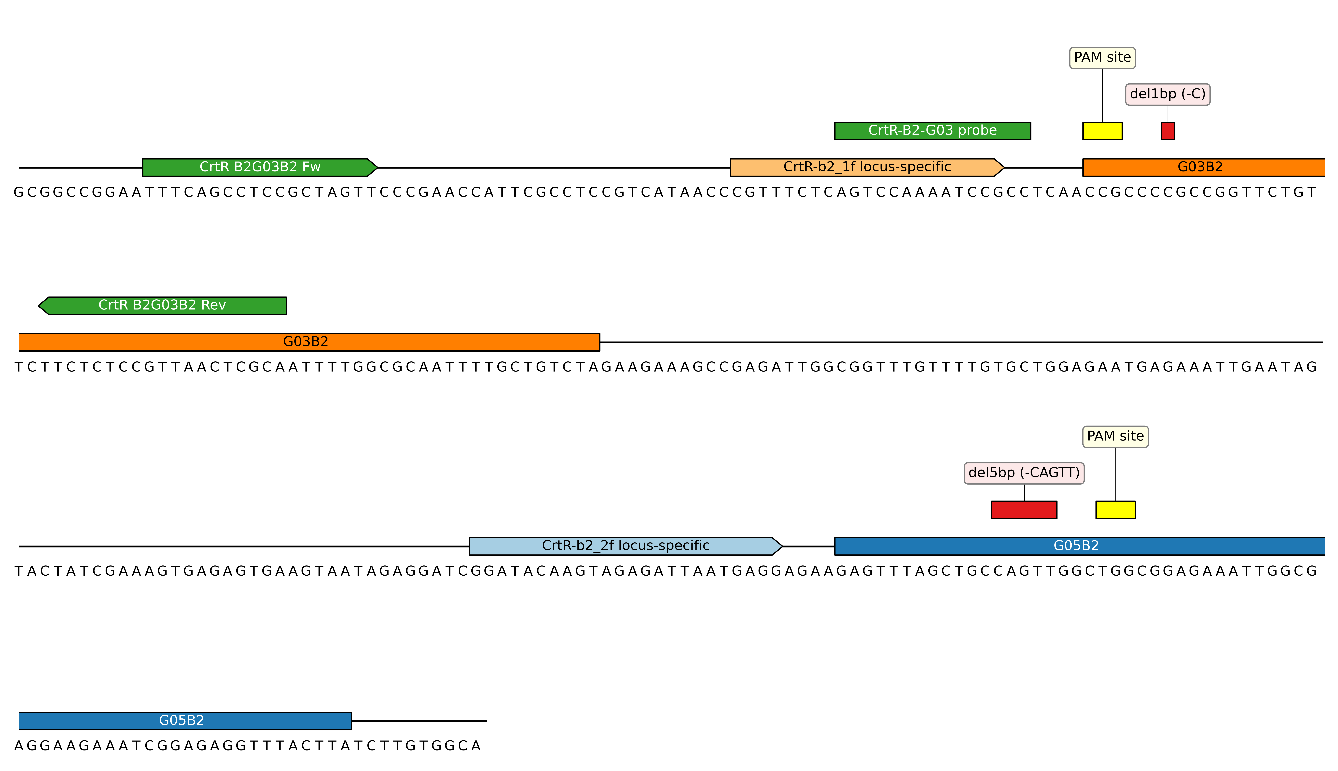


**Figure S2.** Target regions in the tomato CrtR-B2 gene (chromosome SL4.0 Chr03) for the first locus-specific PCR amplification step. Target Loci G03B2 (SL4.0ch03:2472973-2473035) and G05B2 (SL4.0ch03:2473154-2473216) are highlighted respectively in dark orange and blue color. Related primers sequences for the amplification of the two loci are highlighted in light orange and light blue, while the expected mutations are shown in red. Primers and probes for the tomato CrtR-B2 amplification system are designated in green. PAM (protospacer adjacent motif) sites are indicated in yellow colour.


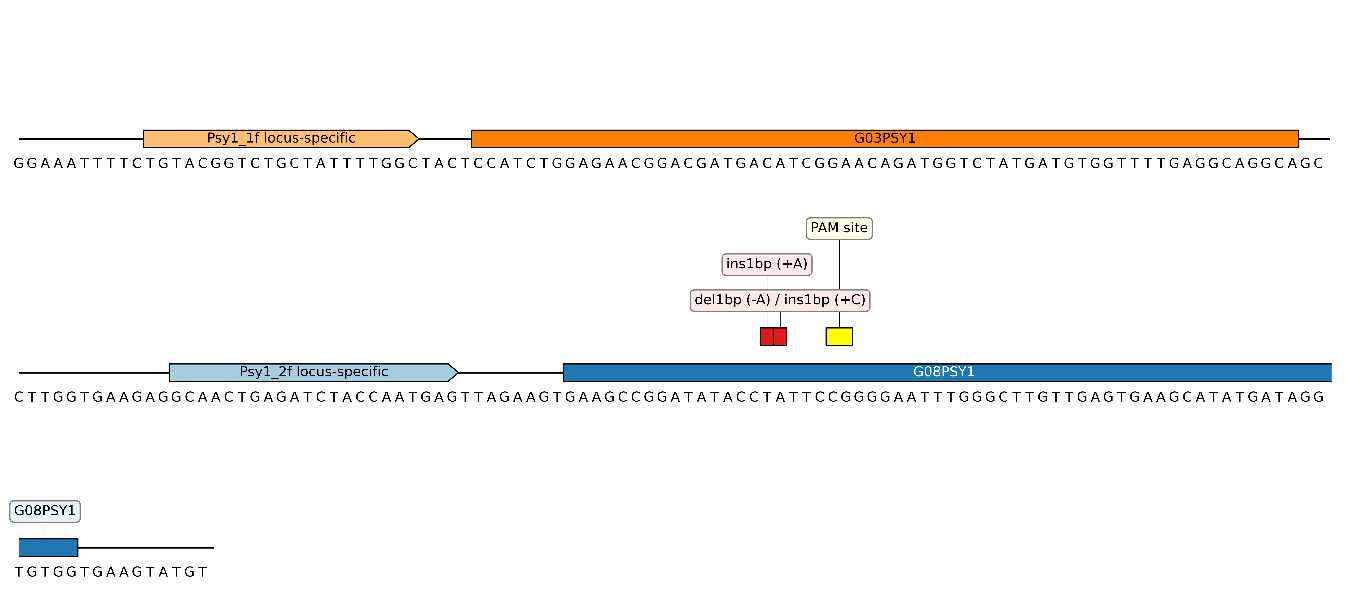


**Figure S3.** Target regions in the tomato Psy1 gene (chromosome SL4.0 Chr03) for the first locus-specific PCR amplification step. Target Loci G03PSY1 (SL4.0ch03:4235230-4235292) and G08PSY1 (SL4.0ch03:4235337-4235399) are highlighted respectively in dark orange and blue color. Related primers sequences for the amplification of the two loci are highlighted in light orange and light blue, while the expected mutations are shown in red. PAM (protospacer adjacent motif) sites are indicated in yellow colour.

| **Universal primer** | **Sequence** | **Length (bp)** | **Tm** |
| --- | --- | --- | --- |
| IL-2 | AATGATACGGCGACCACCGAGATCTACACTCTTTCCCTACACGACGCTCTTCCGATCT | 58 | 84°C |

| **SPE primers** | ***Loci*** | **Sequence** | **Length (bp)** | **Tm** |
| --- | --- | --- | --- | --- |
| CrtR-b2_1f | G03B2 | GTGACTGGAGTTCAGACGTGTGCTCTTCCGATCTAATGTACAGTATTGCGTTTTGCGTTTCTCAGTCCAAAATC*C*G | 76 | 82°C |
| CrtR-b2_2f | G05B2 | GTGACTGGAGTTCAGACGTGTGCTCTTCCGATCTAATGTACAGTATTGCGTTTTGGGATACAAGTAGAGATTAATGA*G*G | 79 | 81°C |
| Psy1_1f | G03PSY1 | GTGACTGGAGTTCAGACGTGTGCTCTTCCGATCTAATGTACAGTATTGCGTTTTGTGTACGGTCTGCTATTTTG*G*C | 76 | 82°C |
| Psy1_2f | G08PSY1 | GTGACTGGAGTTCAGACGTGTGCTCTTCCGATCTAATGTACAGTATTGCGTTTTGGCAACTGAGATCTACCAATG*A*G | 77 | 82°C |

**Table S6.** Universal and SPE primers for the first locus-specific PCR amplification. Sequences of the universal and target primers for the first locus-specific PCR amplification step. The universal sequence highlighted in purple in the Illumina IL-2 primer binds to the duplex adapter. The target primer sequences are made of a universal part of the Illumina adapter (red), a common linker specific to this protocol (blue) and the locus-specific sequences for amplifying the region carrying the mutation (green). * Phosphorothioated DNA bases: This modification renders the inter nucleotide linkage resistant to nuclease degradation.

| **Index TSUDI_10 bp i5 primer** | **Sequence** | **Index TSUDI_10 bp i7 primer** | **Sequence** |
| --- | --- | --- | --- |
| T5_41-p1-A06 | AATGATACGGCGACCACCGAGATCTACACAGTCAGCTGAACACTCTTTCCCTACACGAC | B7_41-p1-A06 | **CAAGCAGAAGACGGCATACGAGAT**ACGGTCTGGAGTGACTGGAGTTCAGACGTGT |
| T5_49-p1-A07 | AATGATACGGCGACCACCGAGATCTACACGAAGACGATAACACTCTTTCCCTACACGAC | B7_49-p1-A07 | **CAAGCAGAAGACGGCATACGAGAT**TTGGCGCTATGTGACTGGAGTTCAGACGTGT |
| T5_57-p1-A08 | AATGATACGGCGACCACCGAGATCTACACATAGTATCGGACACTCTTTCCCTACACGAC | B7_57-p1-A08 | **CAAGCAGAAGACGGCATACGAGAT**TCCGTAAGTAGTGACTGGAGTTCAGACGTGT |
| T5_65-p1-A09 | AATGATACGGCGACCACCGAGATCTACACTTCCGTGAAGACACTCTTTCCCTACACGAC | B7_65-p1-A09 | **CAAGCAGAAGACGGCATACGAGAT**GTTCCTCATTGTGACTGGAGTTCAGACGTGT |
| T5_73-p1-A10 | AATGATACGGCGACCACCGAGATCTACACGACCTGCTTCACACTCTTTCCCTACACGAC | B7_73-p1-A10 | **CAAGCAGAAGACGGCATACGAGAT**TCCAGTCGATGTGACTGGAGTTCAGACGTGT |
| T5_81-p1-A11 | AATGATACGGCGACCACCGAGATCTACACGAGAACTCCAACACTCTTTCCCTACACGAC | B7_81-p1-A11 | **CAAGCAGAAGACGGCATACGAGAT**CGGATATGAGGTGACTGGAGTTCAGACGTGT |

**Table S7.** Primers for the second universal PCR amplification. Two indexes i5 and i7 are needed to perform pair end sequencing. Index TSUDI_10 bp_i5 primer: In blue i5 Illumina barcode to attach flow cell; in yellow primer annealing locus for PCR amplification; underlined the indexes for each library to run up to 6 different samples together; in green specific sequence for Illumina. Index TSUDI_10 bp_i7 primer: in bold i7 Illumina barcode to attach flow cell; in red locus target primer sequences.

| **Locus-specific PCR amplification** | | |
| --- | --- | --- |
| **Stock** | **Reagents** | **Final Concentration** |
| 5X | Q5 Reaction Buffer | 1X |
| 10mM | dNTPs | 500µM |
| 10µM | IL2 primer | 500nM |
| 2.5µM | CrtR-b2_1f | 25nM |
| 2.5µM | CrtR-b2_2f | 25nM |
| 2.5µM | Psy1_1f | 25nM |
| 2.5µM | Psy1_2f | 25nM |
| 2U/µl | Q5 Hot Start High-Fidelity  DNA Polymerase | 0.02U/µL |
| 5X | Q5 High GC Enhancer | 0.5X |
| 2-5ng/µL | DNA template ligated to duplex-adapter | 100/250ng |
|  | Ultrapure water | to 50 µL final volume |

**Table S8.** Final concentration of the reagents for the locus-specific PCR amplification (PCR1). All reagents were combined on ice.

| **Locus-specific PCR amplification** | | | |
| --- | --- | --- | --- |
| **Conditions** | **T (°C)** | **t (min/sec)** | **Cycles** |
| Hot-start – initial denaturation | 95°C | 3' | 1 |
| Denaturation | 98°C | 20'' | 8 |
| Annealing - extension | 65°C | 10' |  |
| final extension | 72°C | 5' | 1 |

**Table S9.** Locus-specific PCR amplification conditions (PCR1).

| **Universal PCR amplification** | | | |
| --- | --- | --- | --- |
| **Stock** |  | **Reagents** | **Final Concentration** |
| 5X |  | Q5 Reaction Buffer | 1X |
| 10mM |  | dNTPs | 500µM |
| 2.5µM |  | SeqCap index primer mix* | 500nM |
| 2U/µl |  | Q5 Hot Start High-Fidelity  DNA Polymerase (New England Biolabs) | 0.02U/µL |
| 2 ng/µl |  | Purified PCR1 template | 200ng |
|  |  | Ultrapure water | to 50µL final volume |

**Table S10.** Final concentration of the reagents for the PCR-universal amplification step (PCR2). All reagents were combined on ice. * Custom UDI 10 bp SeqCap index primer mix.

| **Universal PCR amplification** | | | |
| --- | --- | --- | --- |
| **Conditions** | **T (°C)** | **t (min/sec)** | **cycles** |
| Hot-start – initial denaturation | 95°C | 13' | 1 |
|  | 98°C | 2' | 1 |
| Denaturation | 98°C | 15'' | 20* |
| Annealing - extension | 60°C | 2' |  |
| Final extension | 72°C | 5' | 1 |

**Table S11.** PCR-universal amplification conditions (PCR2). The number of cycles has been reduced to 16 for ten samples.

| **Product** | **Company** | **Code** | **Reaction** |
| --- | --- | --- | --- |
| DNA Polymerase I | New England Biolabs | M02095 | Duplex annealing and extension |
| Q5 Hot Start High-Fidelity DNA Polymerase | New England Biolabs | M04935 | PCR-locus |
| MinElute PCR Purification Kit | QIAGEN | 28004 | Duplex purification |
| KAPA HyperPrep Kits | Roche | KX8503 | End repair, A-tail & Ligation |
| Agencourt AMPure XP beads | Beckman Coulter Life Sciences | A63881 | DNA purification |
| Agilent High Sensitivity DNA Kit | Agilent | 5067-4626 | Electrophoresis |
| Qubit dsDNA HS Assay Kit | Thermo Fisher Scientific | Q33231 | DNA quantification |
| dNTPs | Thermo Fisher Scientific | 10297018 | PCR Amplification |
| TE buffer | Integrated DNA Technologies (IDT) | 11-05-01-13 | Oligonucleotides resuspension |
| Seq-cap primer mix UDI | Illumina | FC-131-1001 | Attach i7 barcode and remainder Illumina P7 sequence |
| NovaSeq 6000 S4 Reagent Kit v1S | Illumina | 20028312 | Illumina cluster generation and sequencing |
| Nuclease-free water | Thermo Fisher Scientific | 10320995 | DNA purification |
| Ethanol 100% | Sigma-Aldrich | 51976 | DNA purification |

**Table S12.** Reagents used for genomic library preparation and sequencing.


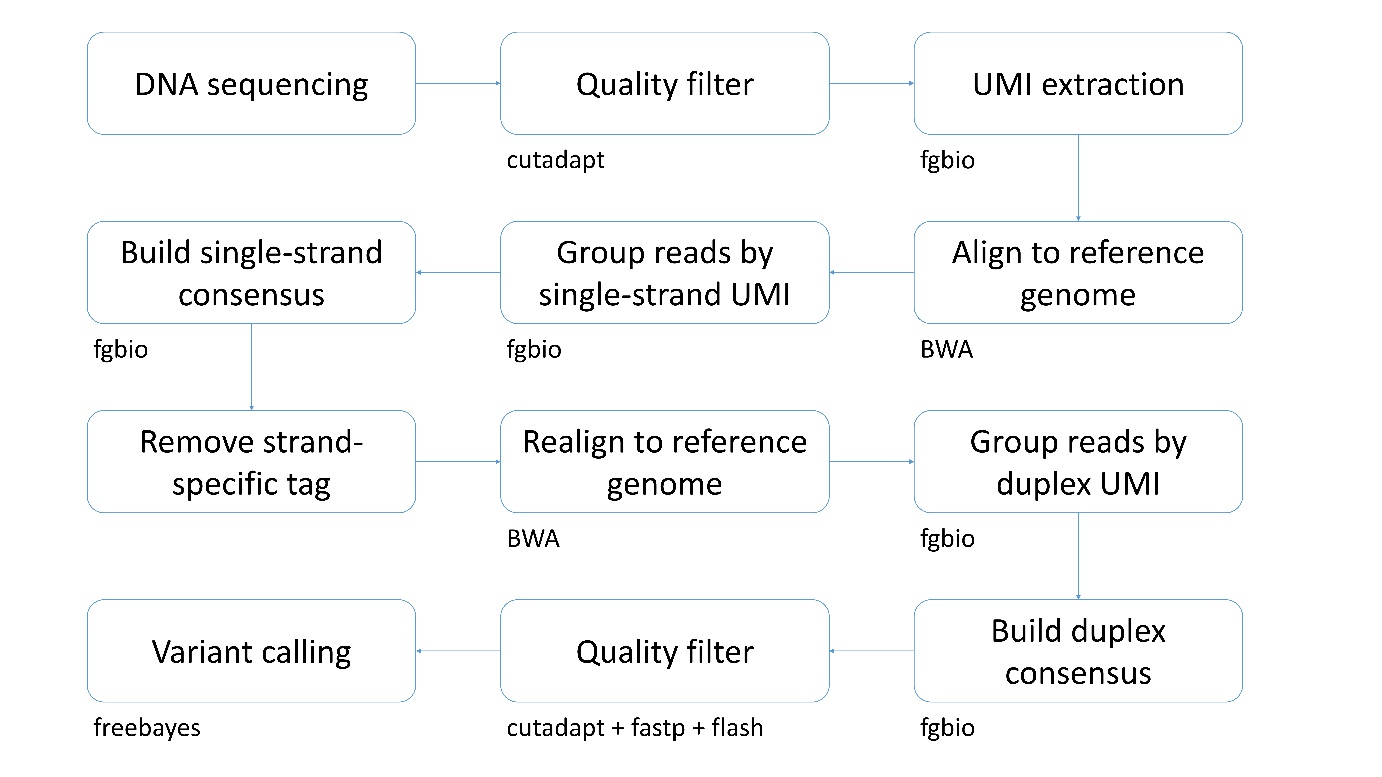


**Figure S4.** Bioinformatics processing workflow for variant identification. 1) DNA sequencing: multiplexed samples from the same sequencing lane were demultiplexed and sorted into separate files according to their index sequence. 2) Quality filter: sequencing reads with length from 50 to 200 bp were extracted using *cutadapt* version 2.6 (parameters: --trim-n --length 200 --minimum-length 50 --pair-filter=any). 3) UMI extraction: the *FastqToBam* tool from the *fgbio* suite (version 1.3.0) was used to identify and extract the UMIs, while converting the sequence files from FASTQ to unmapped BAM format (parameters: -s --read-structures 12M10S2M12S+T 20S+T). 4) Align to reference genome: to obtain the location coordinates that drive UMI grouping, the reads were preliminary mapped on the tomato reference genome SL4.0 (Hosmani et al., 2019), using the *BWA-mem* aligner (version 0.7.17). 5) Group reads by single-strand UMI: the reads were grouped according to their UMI in a strand-specific manner using the *GroupReadsByUmi* tool from the *fgbio* suite, considering only reads with a minimum mapping quality of 20 (parameters: --strategy adjacency --edits 1 –min-map-q 20). 6) Build single-strand consensus: a consensus was built for the reads sharing the same strand-specific UMI using the *CallMolecularConsensusReads* tool from the *fgbio* suite. The consensus reads were then filtered using *FilterConsensusReads* tool also from the *fgbio* suite for a mapping quality of at least 25 for at least 80 % of the consensus sequence (parameters: --reverse-per-base-tags=true --min-reads 1 -E 0.1 -N 25 -e 0.1 -n 0.2). 7) Remove strand-specific tag: strand-specific tags were removed from the UMI. 8) Realign to reference genome: the consensus reads were mapped again on the tomato genome and the alignments were filtered to exclude those with mapping quality lower than 20 using *samtools* version 1.12**.** 9) Group reads by duplex UMI: the sequences were grouped according to their UMI. 10) Build duplex consensus: a second consensus was built, this time across the two strands, when at least one read was available from each DNA strand. The consensus were filtered to retain only those with an error rate below 0.05 and 0.1 respectively at the read and base level, as well as a mapping quality higher or equal to 30 for at least 90% of the consensus sequence (parameters: --reverse-per-base-tags=true --min-reads 1 -E 0.05 -N 30 -e 0.1 -n 0.1). 11) Quality filter: *cutadapt* was used to remove from these consensus sequences primer overhangs (parameters: --discard-untrimmed --pair-filter=first --minimum-length 35:35 --times 3 – overlap 15) and adapter leftovers (parameters: --minimum-length 35:35 --overlap 15 -u -2 --times 3). Another quality-filtering step was performed using *fastp* version 0.20.0 (parameters: --cut_tail --cut_front --cut_tail_mean_quality 30 --cut_front_mean_quality 30 --n_base_limit 20 --disable_adapter_trimming). Potential artefacts generated by counting overlapping reads from paired end sequencing as independent molecules are avoided by using *flash* v.1.2.11 (-M 100) that merges the two overlapping reads from paired end sequencing in one single consensus sequence. 12) Variant calling: variant calling was finally performed using *freebayes* version 1.3.2 with a minimum coverage of two duplex consensus sequences (parameters: --pooled-continuous --min-alternate-fraction 0.0001 --min-alternate-count 2 --use-best-n-alleles 2).


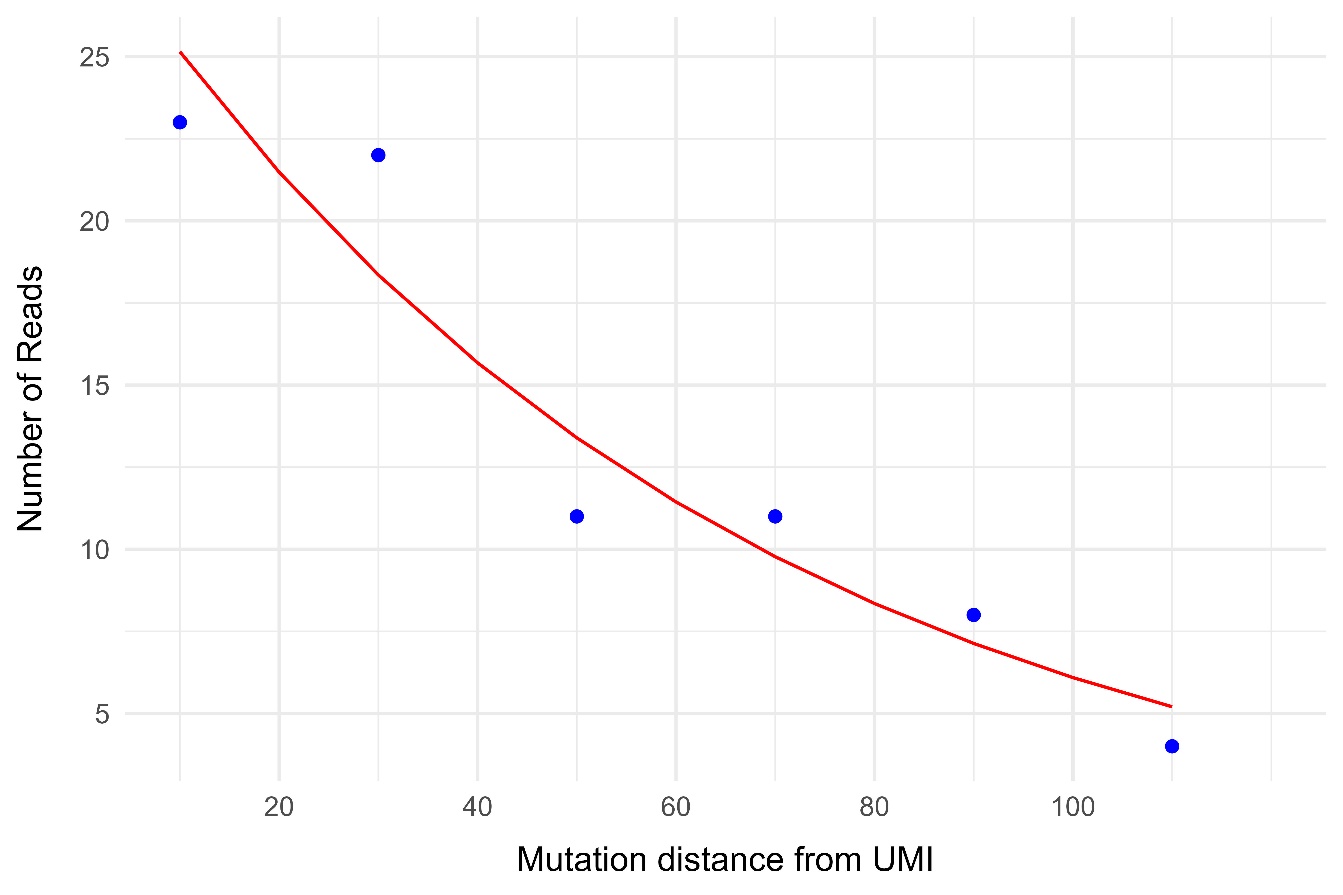


**Figure S5.** Observed and predicted number of reads from the Poisson regression model plotted versus the mutation distance from UMI. The values on the Y-axis indicate the number of duplex consensus reads (response variable) presenting unexpected mutations while the ones on the X-axis their distance from the UMI sequence in base pairs (independent variable). In these analyses, we made use of the midpoint of each bin (e.g., 10 for the 1-20 range of the mutation distance from UMI) containing a specific number of reads. The blue dots represent the observed number of duplex consensus reads presenting unexpected mutations, while the red curve illustrates the Poisson regression model, showing a very good fit of the model to the data. The statistical analysis revealed that the mutation distance from the UMI had a highly significant (p< 0.01) negative effect on the number of reads for the unexpected mutations.

| **Library ID** | **Sequencing reads** | | **Consensus sequences (filtered)** | | **Sequences on target** | | | **UMI representation (%)** | | |  |
| --- | --- | --- | --- | --- | --- | --- | --- | --- | --- | --- | --- |
|  | Raw | Filtered | Single strand | Duplex | CrtR_B2 | Psy1 | Total | 1 read | 2-5 reads | >5 reads |  |
| 0.1A* | 84,679,110 | 84,679,110 | 2,596,612 | 23,099 | 8,976 (39%) | 11,764 (51%) | 20,740 (90%) | 51% | 30% | 19% |  |
| 0.1B* | 74,032,650 | 74,032,650 | 2,654,466 | 26,019 | 10,014 (38%) | 12,917 (50%) | 22,931 (88%) | 50% | 31% | 19% |  |
| 0.1C* | 100,498,204 | 100,498,204 | 3,998,746 | 27,144 | 10,742 (40%) | 13,095 (48%) | 23,837 (88%) | 44% | 32% | 23% |  |
| 0.1_450A | 120,763,722 | 120,763,722 | 3,543,934 | 28,852 | 10,093 (35%) | 18,390 (64%) | 28,483 (99%) | 68% | 23% | 9% |  |
| 0.1_450B | 151,883,850 | 151,883,850 | 4,400,152 | 41,288 | 14,880 (36%) | 25,643 (62%) | 40,523 (98%) | 67% | 22% | 11% |  |
| 0.1_450C* | 130,196,968 | 130,196,968 | 4,570,774 | 57,697 | 24,311 (42%) | 26,413 (46%) | 50,724 (88%) | 48% | 31% | 20% |  |
| 0.5A | 171,281,124 | 171,281,124 | 3,513,086 | 23,739 | 7,954 (34%) | 14,021 (59%) | 21,975 (93%) | 65% | 23% | 12% |  |
| 0.5B* | 59,900,024 | 59,900,024 | 2,455,268 | 21,300 | 7,984 (37%) | 10,434 (49%) | 18,418 (86%) | 47% | 36% | 17% |  |
| 0.5C* | 50,733,142 | 50,733,142 | 2,185,814 | 21,261 | 8,037 (38%) | 10,397 (49%) | 18,434 (87%) | 48% | 36% | 16% |  |
| 0.9A | 180,406,476 | 180,406,476 | 2,496,918 | 18,194 | 6,327 (35%) | 10,830 (60%) | 17,157 (94%) | 67% | 22% | 11% |  |
| 0.9B | 138,672,338 | 138,672,338 | 2,684,244 | 21,685 | 8,139 (38%) | 12,404 (57%) | 20,543 (95%) | 65% | 23% | 13% |  |
| 0.9C* | 67,248,268 | 67,248,268 | 2,646,356 | 25,005 | 9,685 (39%) | 11,991 (48%) | 21,676 (87%) | 47% | 35% | 18% |  |
| 10A | 139,121,762 | 139,121,762 | 2,940,684 | 21,001 | 6,969 (33%) | 13,474 (64%) | 20,443 (97%) | 67% | 22% | 10% |  |
| 10B | 143,467,538 | 143,467,538 | 2,758,228 | 22,226 | 7,775 (35%) | 13,420 (60%) | 21,195 (95%) | 66% | 22% | 12% |  |
| 10C | 130,006,186 | 130,006,186 | 2,376,692 | 19,618 | 6,848 (35%) | 12,166 (62%) | 19,014 (97%) | 68% | 21% | 11% |  |
| Crt_Δ1 | 159,998,186 | 159,066,136 | 2,367,612 | 20,599 | 8,022 (39%) | 11,788 (57%) | 19,810 (96%) | 66% | 22% | 12% |  |
| Crt_Δ5 | 160,206,318 | 159,233,284 | 2,879,700 | 24,811 | 9,445 (38%) | 14,383 (58%) | 23,828 (96%) | 65% | 24% | 12% |  |
| Psy_±1 | 62,830,902 | 62,340,558 | 1,640,750 | 17,639 | 6,114 (35%) | 11,240 (64%) | 17,354 (98%) | 64% | 22% | 13% |  |
| Psy_+1* | 34,388,920 | 34,278,322 | 1,354,986 | 20,460 | 7,756 (38%) | 10,679 (52%) | 18,435 (90%) | 51% | 33% | 17% |  |
| WT_A | 144,476,776 | 144,476,776 | 3,099,598 | 24,892 | 8,806 (35%) | 14,943 (60%) | 23,749 (95%) | 67% | 22% | 11% |  |
| WT_B* | 58,404,644 | 58,404,644 | 2,796,014 | 29,112 | 11,254 (39%) | 14,797 (51%) | 26,051 (89%) | 47% | 34% | 19% |  |
| WT_C* | 58,096,562 | 58,096,562 | 1,999,146 | 22,405 | 8,357 (37%) | 11,296 (50%) | 19,653 (88%) | 48% | 35% | 17% |  |
| Average | 110,058,803 | 109,944,893 | 2,816,354 | 25,366 | 9,477 (37%) | 14,090 (56%) | 23,408 (93%) | 58% | 27% | 15% |  |
|  |  |  |  |  |  |  |  |  |  |  |  |

**Table S13.** Sequencing reads analysis in the Psy1 and GrtR-B2 target regions for all samples. Results for all samples (Library ID) at the different sequence analysis steps. In the different columns are indicated the number of raw and filtered sequencing reads, the single strand and duplex consensus sequences, the sequences on NGT target regions, and the average number of reads for UMI molecule. The analysis on the error rates considered only the NGT regions of interest (ROI) and the alignments with decent mapping quality (MAPQ>20). The reads were calculated at two stages: 1) Raw data (without considering the UMIs) before and after filtering; 2) Consensus sequences: the single-strand consensus reads, built after grouping reads with the same UMI - but no duplex) and duplex (i.e. the final duplex consensus reads, built after grouping reads with the same UMI in the two strands). WT= wild type *S. lycopersicum* cv *Red Setter* tomato line. * Refers to libraries that were amplified with a reduction to 16 cycles in the second PCR enrichment step.


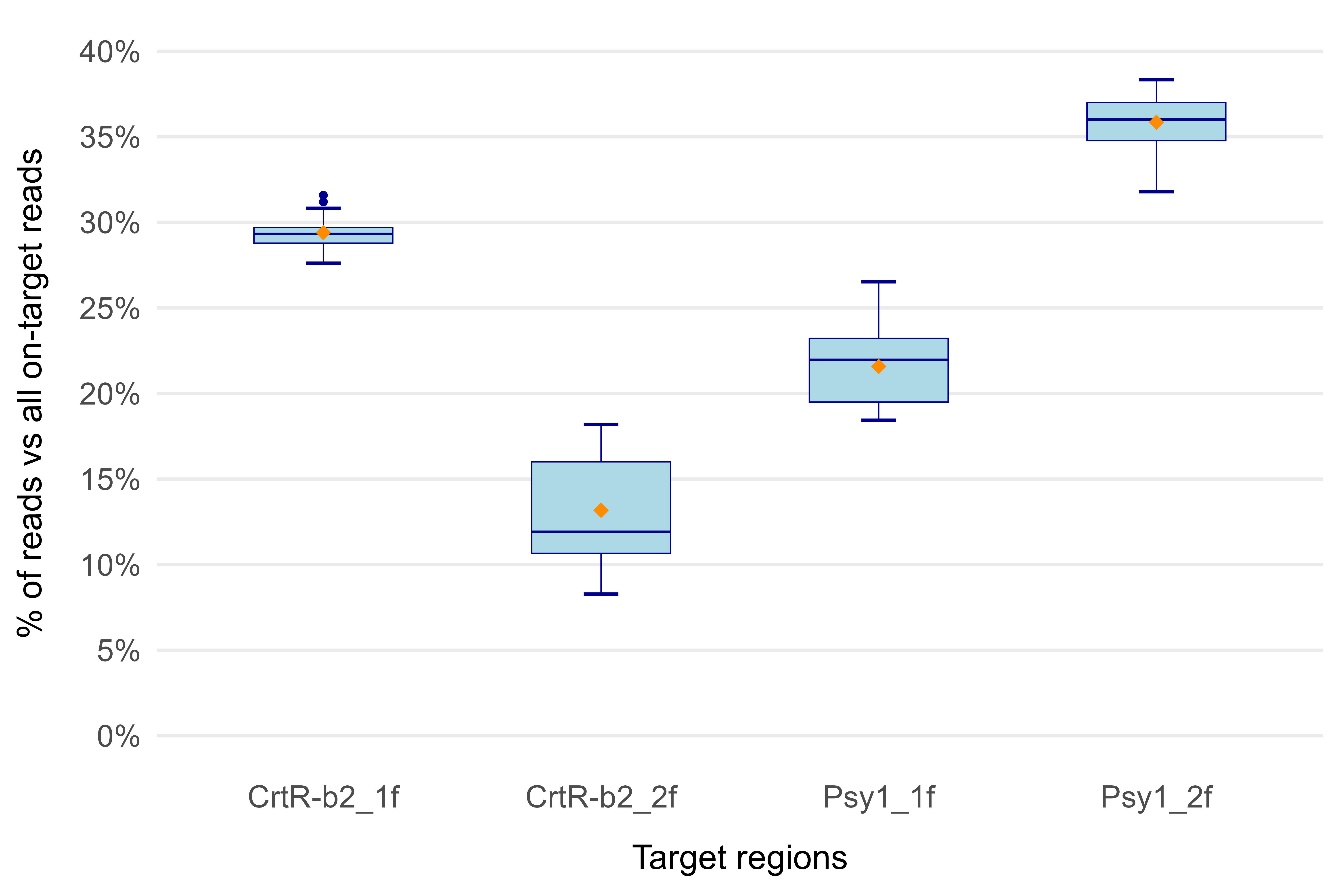


**Figure S6.** Percentage on-target reads for each different locus-specific primer (SPE). CrtR-b2_1f and CrtR-b2_2f primers amplify respectively the G03B2 and G05B2 NGT target regions in the CrtR-B2 gene (Figure S1 in Supplementary Annex). Psy1_1f and Psy1_2f primers amplify respectively the G03PSY1 and G08PSY1 NGT target regions in the Psy1 gene (Figure S2 in Supplementary Annex). The blue line and the orange dot in the box indicate the median and the average value of the data set, respectively.

| **ROI** | **Start Position** | **End Position** | **WT Seq.** | **Mut. Seq.** | **WT_A** | **WT_B** | **WT_C** | **Psy_+1** | **Crt_Δ1** | **Crt_Δ5** | **Psy_±1** |
| --- | --- | --- | --- | --- | --- | --- | --- | --- | --- | --- | --- |
| G03B2  (CrtR-B2) | 2472975 | 2472976 | GC | G |  |  |  |  | 100.000 |  |  |
|  | 2472980 | 2472980 | G | T |  |  |  |  |  |  | 0.062 |
|  | 2473009 | 2473009 | G | T |  |  |  |  | 0.066 |  |  |
|  | 2473020 | 2473020 | G | A |  |  |  |  | 0.036 |  |  |
|  | 2473023 | 2473023 | A | G |  |  |  |  | 0.037 |  |  |
|  | 2473024 | 2473024 | T | A |  |  |  |  | 0.036 |  |  |
|  | 2473028 | 2473028 | G | A |  |  |  |  | 0.037 |  |  |
| G05B2  (CrtR-B2) | 2473165 | 2473165 | C | A* |  |  |  | 0.150 |  |  |  |
|  | 2473165 | 2473170 | CCAGTT | C |  |  |  |  |  | 99.936 |  |
|  | 2473195 | 2473195 | A | G* |  |  |  |  |  | 0.547 |  |
|  | 2473197 | 2473197 | A | G* |  |  |  |  |  | 0.385 |  |
|  | 2473201 | 2473201 | C | A* |  |  |  |  |  |  | 0.279 |
|  | 2473201 | 2473201 | C | CA* |  |  |  |  |  | 0.526 |  |
|  | 2473208 | 2473208 | G | A* |  |  |  |  |  | 0.215 |  |
|  | 2473209 | 2473209 | T | A* |  |  |  |  |  | 0.525 |  |
|  | 2473213 | 2473213 | C | G |  |  | 0.096 |  |  |  |  |
| G03PSY  (Psy1) | 4235268 | 4235268 | C | A |  |  |  |  | 0.066 |  |  |
|  | 4235278 | 4235278 | G | T |  |  |  |  | 0.046 |  |  |
| G08PSY1  (Psy1) | 4235341 | 4235341 | C | A | 0.039 |  |  |  |  | 0.026 |  |
|  | 4235351 | 4235351 | C | T |  |  |  | 0.051 |  |  |  |
|  | 4235352 | 4235352 | T | TA |  | 0.049 | 0.098 | 99.151 |  | 0.070 |  |
|  | 4235352 | 4235353 | TA | T |  |  |  |  |  |  | 54.660 |
|  | 4235353 | 4235353 | A | AC |  |  |  |  |  |  | 45.262 |
|  | 4235370 | 4235370 | C | A |  |  | 0.063 |  |  |  | 0.030 |
|  | 4235384 | 4235384 | C | A |  |  |  |  |  |  | 0.033 |

**Table S14.** Mutation frequency detected for the wild type (WT_A, WT_B and WT_C) and pure NGT lines (Psy_+1, Crt_Δ1, Crt_Δ5 and Psy_±1). The first column indicates the gene and the region of interest (ROI) of the detected mutation. The four following columns provide the initial and end position of the wild-type sequence (WT Seq.) and corresponding NGT mutation (Mut. Seq.). The other columns represent the sequenced wild type and NGT samples. Horizontal lines highlighted in darker green report the frequencies of expected mutations detected from homozygous NGT lines while those in lighter green report data from the heterozygous NGT line; the white horizontal lines report the frequency of unexpected mutations. Unexpected mutations with an asterisk * were detected at a frequency equal or higher than 0.1 %.


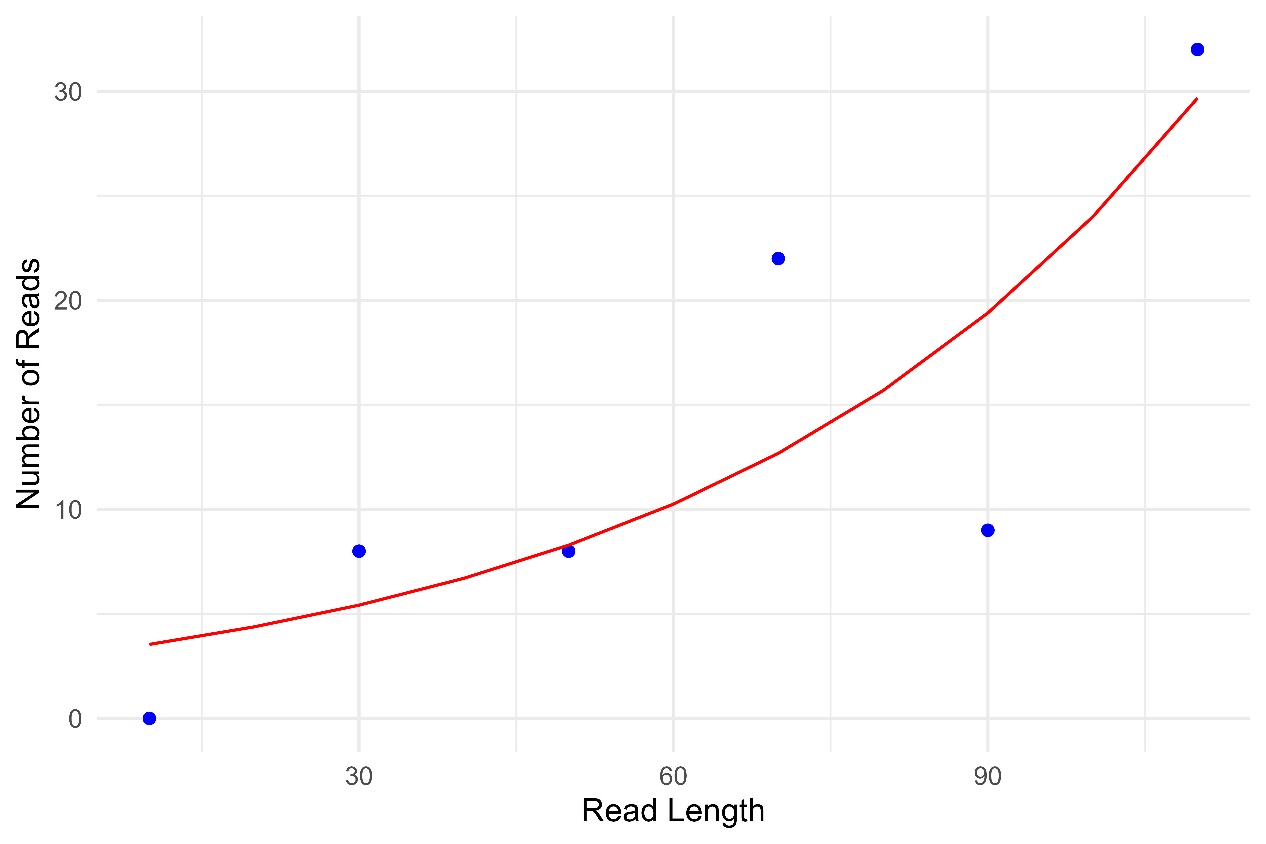


**Figure S7.** Distribution of read lengths of the unexpected mutations. Distribution of duplex consensus reads according to their length. The data indicate that the number of mutated reads increase with the corresponding read length.

**
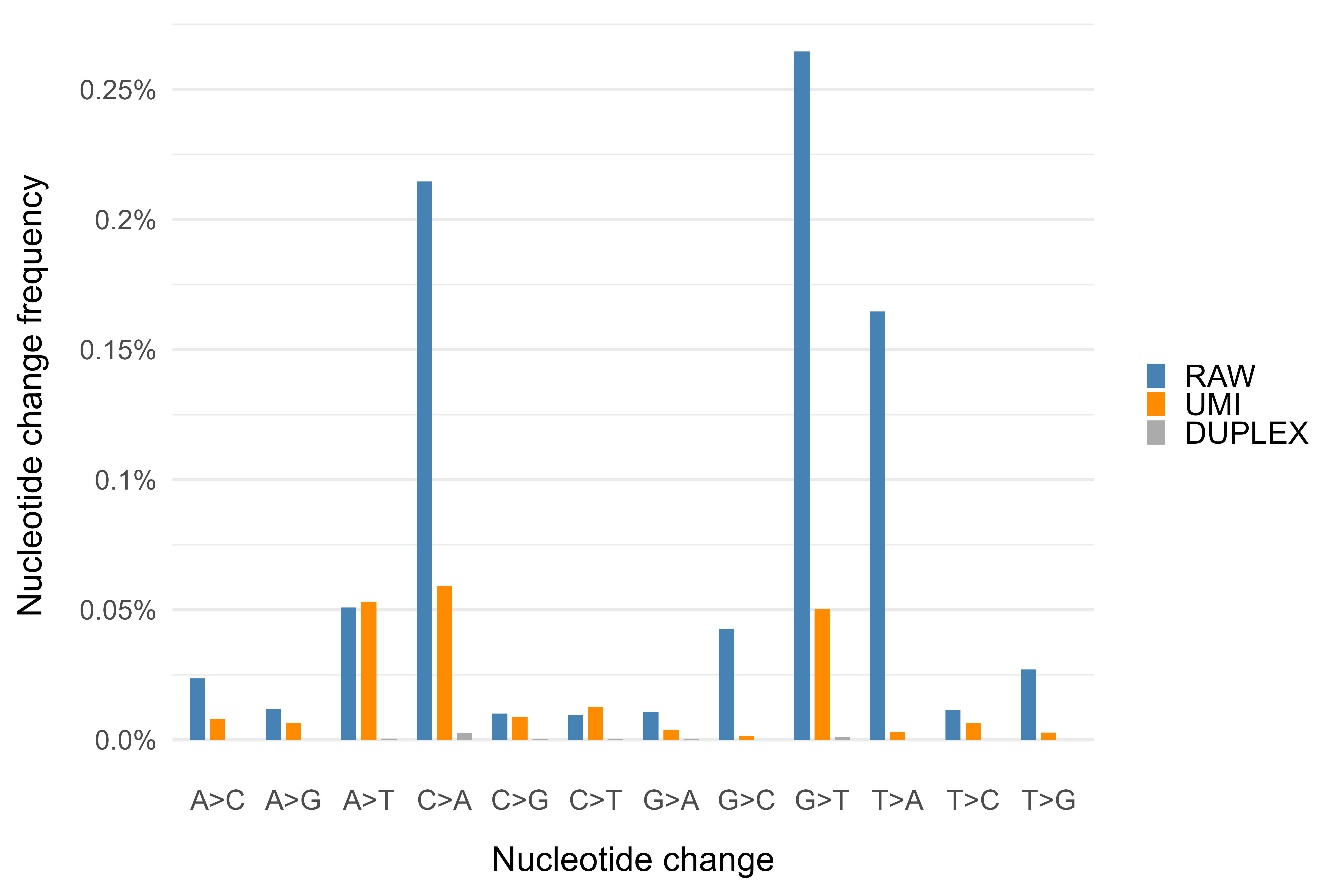
**

**Figure S8.** Frequency of nucleotide changes compared to the reference genome, at different stages of the bioinformatics workflow (raw reads (blue), single UMI consensus reads (orange), duplex consensus reads (grey)). The duplex reads do not always show up in the graph since their value is more than two orders of magnitude lower in frequency of variant bases compared to that of raw reads which include mostly sequencing errors (raw reads)) or PCR miss-incorporation (simplex UMI).


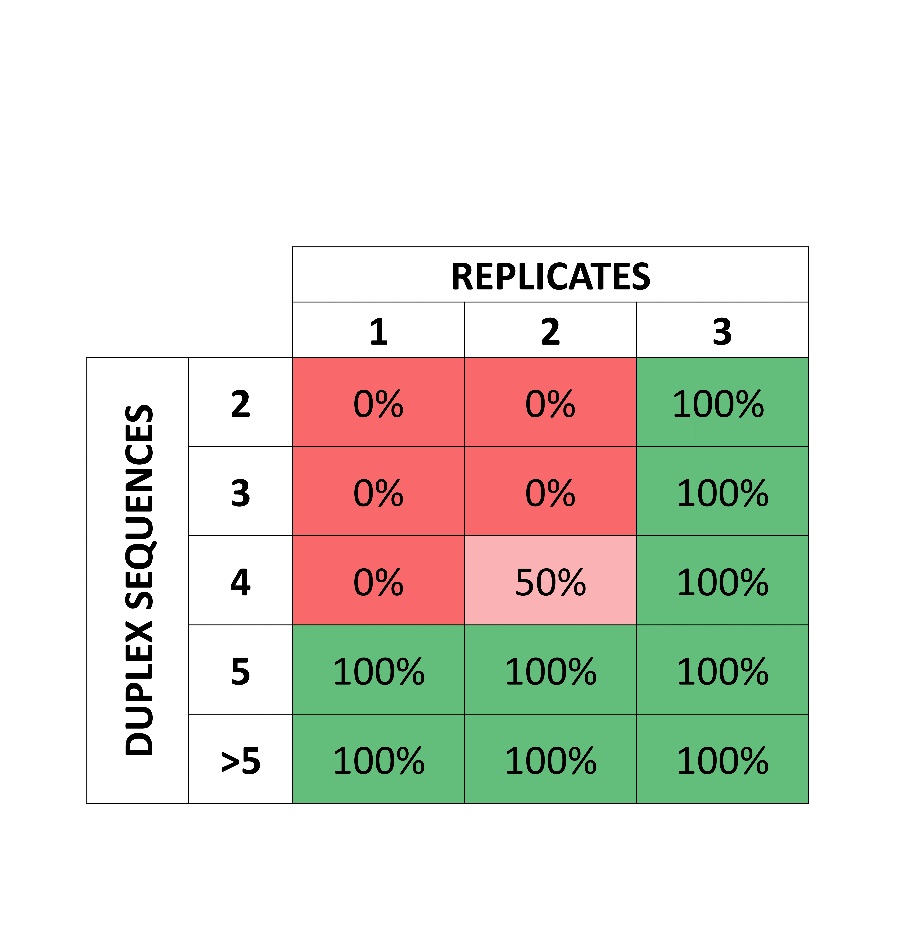


**Figure S9.** True discovery rate. Percentage of expected mutations calls compared to the total number of mutation calls in the 0.1 %, 0.5 %, 0.9 % and 10 % NGT spike-in samples, for each combination of number of replicates (1, 2 and 3) and number of duplex sequences (2, 3, 4, 5 and >5). Box highlighted in red represent combinations of number of duplex molecules and replicates that may provide false positive un-expected mutations (0 % true discovery rate), Box highlighted in green represent combinations of number of duplex molecules and replicates that provide expected real positive NGT mutations (100 % true discovery rate).

**
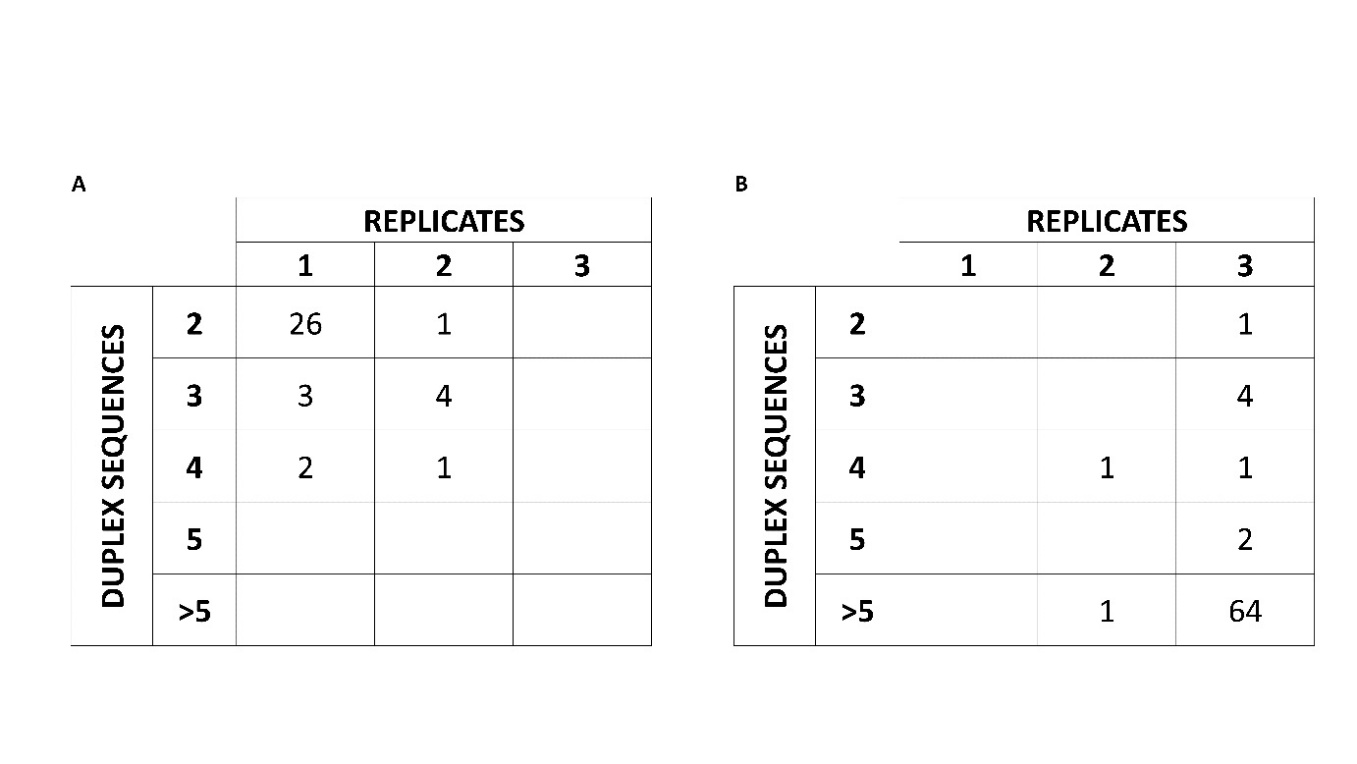
**

**Figure S10.** True discovery rate of unexpected mutations versus expected mutations. Number of calls for unexpected and expected mutations in the 0.1 %, 0.5 %, 0.9 %, and 10 % samples grouped by the number of replicates in which the mutation was called and by the number of duplex consensus sequences supporting the call. Note: if the same genomic mutation was called in two or three replicates, it will be counted two or three times respectively in the matrix.

| **Library sample** | **5 M** | **10 M** | **25 M** | **50 M** | **AVE** | **SD** |
| --- | --- | --- | --- | --- | --- | --- |
| 0.1A | 0.174% | 0.156% | 0.149% |  | 0.160% | 0.013% |
| 0.1B | 0.112% | 0.109% | 0.120% |  | 0.113% | 0.006% |
| 0.1C | 0.139% | 0.143% | 0.143% |  | 0.142% | 0.003% |
| 0.1_450A | 0.141% | 0.149% | 0.131% | 0.137% | 0.140% | 0.007% |
| 0.1_450B | 0.136% | 0.131% | 0.127% | 0.119% | 0.128% | 0.007% |
| 0.1_450C | 0.117% | 0.112% | 0.111% | 0.109% | 0.112% | 0.003% |

**Table S15.** Distribution frequency of duplex molecules/coverage. Results of the permutation/titration analysis for defining the minimal coverage at the 0.1 % level. Average detection frequency (AVE) and standard deviation (SD) per library sample (0.1A, 0.1B and 0.1C and 0.1_450A, 0.1_450B and 0.1_450C) at different millions reads coverage (5, 10, 25 and 50 M) for all tomato lines.


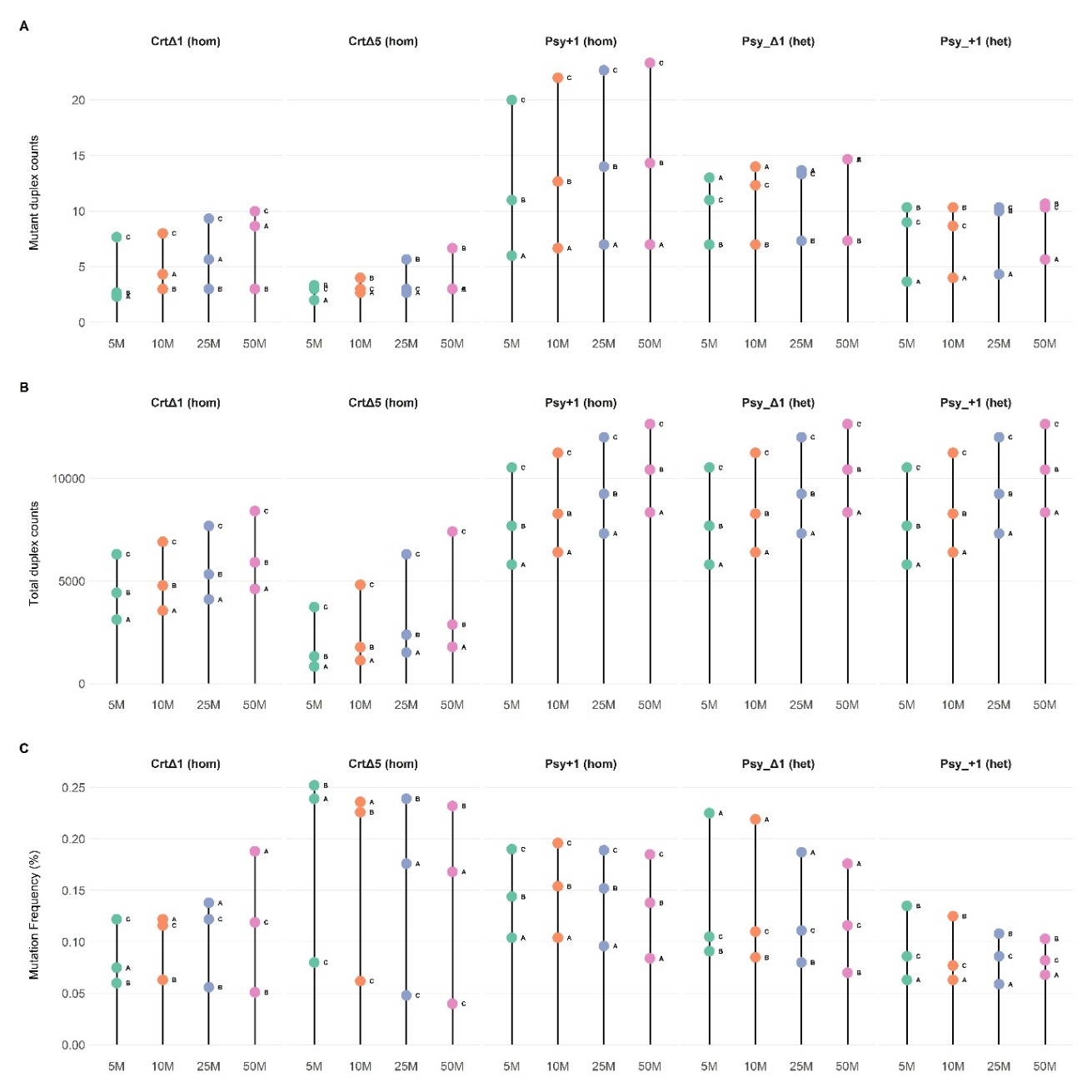


**Figure S11.** Average mutant duplex counts (A), total duplex counts (B) and mutation frequency (C) from the three replicates at the different down-sampling levels plotted versus line-specific mutations. Statistical methods more appropriate for discrete numbers such as counts rather than their frequencies were applied for the analysis since the mutant duplex counts have very low values (ranging from 2 to 24). Here we used Poisson regression for the statistical assessment. The average counts from the different down-sampling levels (5M, 10M, 25M and 50M) are distinguished by different colors. For each line-specific mutation and subsampling level, the three dots with the same color represent data originating from three different libraries. Psy1_+1 (het) and Psy_Δ1 (het) are two alleles of the same heterozygous line Psy_±1. The results suggest an impact of the total duplex counts on the average mutant duplex counts. The selected statistical model has therefore been adjusted to account for the total number of counts per experiment.

**
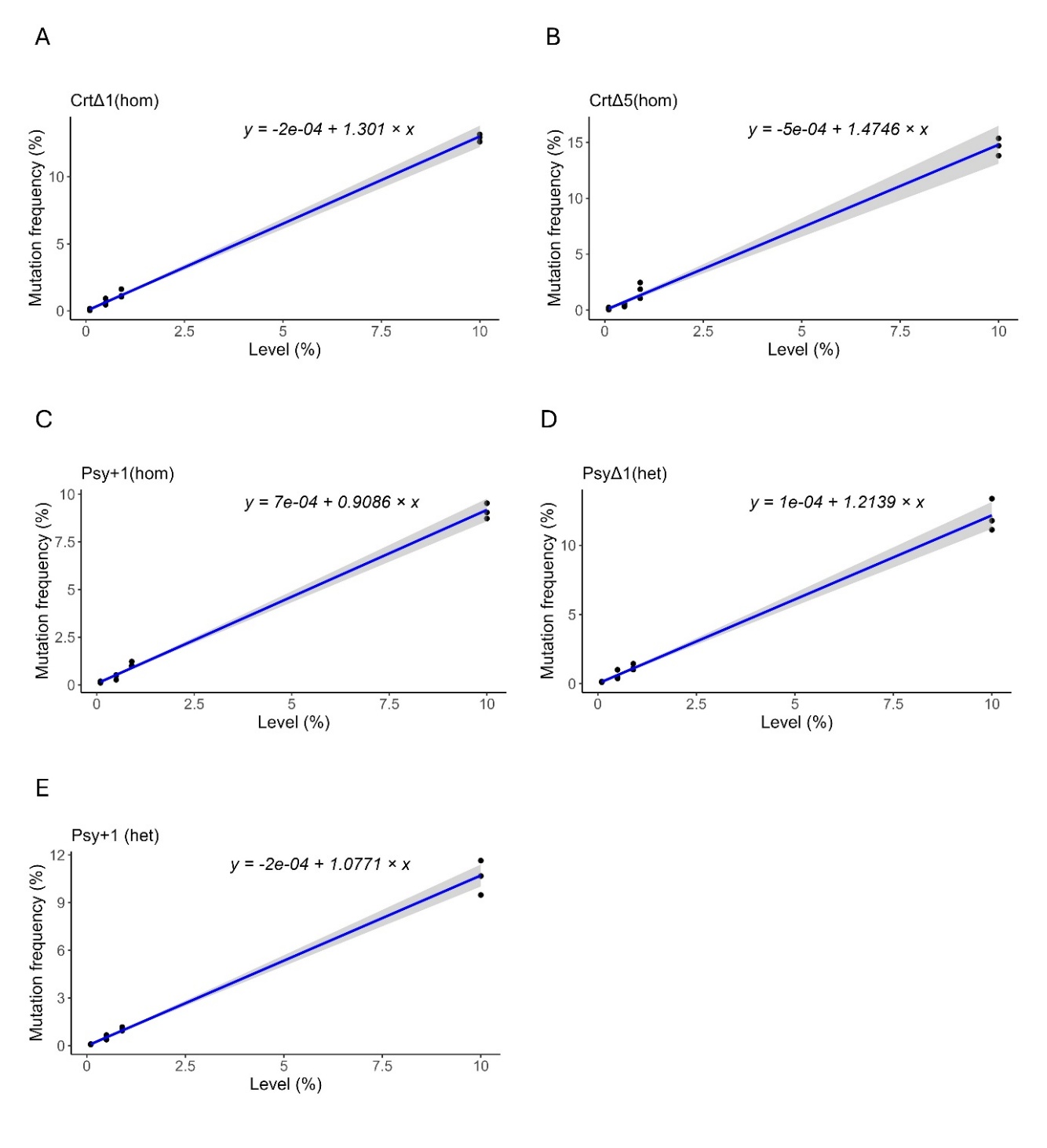
**

**Figure S12.** Relationship between the mutation frequency and the level (copy number ratio). The axes are expressed as percentages (%), but the fitted regression equation shown on the plot is based on the original values ranging from 0 to 1, as used in the statistical model. Psy1_+1 (het) and Psy_Δ1 (het) are two alleles of the same heterozygous line Psy_±1.

| **Libraries** | **Parameter** | **Mutation ID** | | | | | **Grand mean** |
| --- | --- | --- | --- | --- | --- | --- | --- |
|  |  | **CrtΔ1 (hom)** | **CrtΔ5 (hom)** | **Psy+1 (hom)** | **Psy_Δ 1 (het)** | **Psy_+1 (het)** |  |
| **0.1A** | Mutation frequency (%) | 0.21 | 0.14 | 0.18 | 0.08 | 0.13 |  |
| **0.1B** |  | 0.09 | 0.26 | 0.03 | 0.18 | 0.04 |  |
| **0.1C** |  | 0.10 | n.d. | 0.13 | 0.16 | 0.09 |  |
| **0.1_ABC** | Mean mutation frequency (%) | 0.13 | 0.2 | 0.11 | 0.14 | 0.09 | **0.13** |
|  | Bias (%) | 34 | 101 | 12 | 39 | -13 | **34** |
|  | CV (%) | 49 | 41 | 67 | 37 | 49 | **49** |
| **0.1_450A** | Mutation frequency (%) | 0.17 | 0.16 | 0.11 | 0.16 | 0.06 |  |
| **0.1_ 450B** |  | 0.04 | 0.24 | 0.19 | 0.10 | 0.10 |  |
| **0.1_450C** |  | 0.12 | 0.04 | 0.17 | 0.11 | 0.07 |  |
| **0.1_450ABC** | Mean mutation frequency (%) | 0.11 | 0.15 | 0.16 | 0.12 | 0.08 | **0.12** |
|  | Bias (%) | 11 | 45 | 56 | 23 | -23 | **22** |
|  | CV (%) | 60 | 71 | 28 | 26 | 27 | **42** |
| **0.5A** | Mutation frequency (%) | 0.46 | 0.62 | 0.48 | 0.48 | 0.61 |  |
| **0.5B** |  | 0.93 | 0.30 | 0.52 | 0.38 | 0.38 |  |
| **0.5C** |  | 0.6 | 0.45 | 0.27 | 1.00 | 0.66 |  |
| **0.5_ABC** | Mean mutation frequency (%) | 0.67 | 0.45 | 0.42 | 0.62 | 0.55 | **0.54** |
|  | Bias (%) | 33 | -9.0 | 16 | 24 | 9.8 | **15** |
|  | CV (%) | 36 | 35 | 33 | 54 | 28 | **37** |
| **0.9A** | Mutation frequency (%) | 1.1 | 1.1 | 1.2 | 1.0 | 0.9 |  |
| **0.9B** |  | 1.6 | 2.5 | 1.0 | 1.1 | 1.1 |  |
| **0.9C** |  | 1.1 | 1.9 | 1.0 | 1.4 | 1.2 |  |
| **0.9_ABC** | Mean mutation frequency (%) | 1.3 | 1.8 | 1.1 | 1.2 | 1.1 | **1.3** |
|  | Bias (%) | 41 | 100 | 19 | 33 | 17 | **42** |
|  | CV (%) | 24 | 39 | 13 | 19 | 11 | **21** |
| **10A** | Mutation frequency (%) | 13 | 15 | 9.1 | 11 | 12 |  |
| **10B** |  | 13 | 14 | 8.7 | 12 | 11 |  |
| **10C** |  | 13 | 15 | 9.5 | 13 | 9.5 |  |
| **10_ABC** | Mean mutation frequency (%) | 13 | 15 | 9.1 | 12 | 11 | **12** |
|  | Bias (%) | 29 | 46 | -8.9 | 21 | 6.1 | **19** |
|  | CV (%) | 2.1 | 5.3 | 4.5 | 9.6 | 10 | **6.3** |
|  | **Grand mean bias (%)** | **29** | **46** | **21** | **25** | **2.5** |  |
|  | **Grand mean CV (%)** | **31** | **38** | **20** | **27** | **19** |  |

**Table S16.** Mean values of trueness (bias %) and precision (Coefficient of Variation CV %) for the different line-specific NGT mutations and the spike-in triplicates (0.1ABC; 0.1_450ABC; 0.5ABC; 0.9ABC; 10ABC). For each locus, the relative abundance of the mutant NGT allele (Mut. %) was measured as the ratio of the number of mutant duplex consensus sequences carrying the same exact mutation to the total number of duplex consensus sequences and averaged across the three replicates. The numerical values were rounded keeping two digits for values ≤ 1, one digit for values between 1 and 10 and no digits for values ≥ 10. CV % = coefficient of variation; hom= homozygous line; het= heterozygous line; n.d. = not determined because values were available from only two replicates out of three. Psy1_+1 (het) and Psy_Δ1 (het) are two alleles of the same heterozygous line Psy_±1
